# The paracaspase MALT1 cleaves HOIL1 reducing linear ubiquitination by LUBAC to dampen lymphocyte NF-κB signalling

**DOI:** 10.1038/ncomms9777

**Published:** 2015-11-03

**Authors:** Theo Klein, Shan-Yu Fung, Florian Renner, Michael A. Blank, Antoine Dufour, Sohyeong Kang, Madison Bolger-Munro, Joshua M. Scurll, John J. Priatel, Patrick Schweigler, Samu Melkko, Michael R. Gold, Rosa I. Viner, Catherine H. Régnier, Stuart E. Turvey, Christopher M. Overall

**Affiliations:** 1 Department of Biochemistry and Molecular Biology, University of British Columbia, Vancouver, British Columbia, Canada V6T 1Z3; 2 Department of Oral Biological and Medical Science, University of British Columbia, Vancouver, British Columbia, Canada V6T 1Z3; 3 Center for Blood Research, University of British Columbia, Vancouver, British Columbia, Canada V6T 1Z3; 4 Department of Pediatrics, University of British Columbia, Vancouver, British Columbia, Canada V6T 1Z3; 5 Child & Family Research Institute, BC Children's Hospital, Vancouver, British Columbia, Canada V6T 1Z3; 6 Novartis Institutes for BioMedical Research, Novartis Campus, Basel, CH-4056, Switzerland; 7 Thermo Fisher Scientific, 355 River Oaks Parkway, San Jose, 95134 California, USA; 8 Department of Pathology and Laboratory Medicine, University of British Columbia, Vancouver, British Columbia, Canada V5Z 4H4; 9 Department of Microbiology and Immunology, University of British Columbia, Vancouver, British Columbia, Canada V6T 1Z3; 10 Department of Mathematics, University of British Columbia, Vancouver, British Columbia, Canada V6T 1Z3

## Abstract

Antigen receptor signalling activates the canonical NF-κB pathway via the CARD11/BCL10/MALT1 (CBM) signalosome involving key, yet ill-defined roles for linear ubiquitination. The paracaspase MALT1 cleaves and removes negative checkpoint proteins, amplifying lymphocyte responses in NF-κB activation and in B-cell lymphoma subtypes. To identify new human MALT1 substrates, we compare B cells from the only known living *MALT1*^*mut/mut*^ patient with healthy *MALT1*^+/*mut*^ family members using 10-plex Tandem Mass Tag TAILS N-terminal peptide proteomics. We identify HOIL1 of the linear ubiquitin chain assembly complex as a novel MALT1 substrate. We show linear ubiquitination at B-cell receptor microclusters and signalosomes. Late in the NF-κB activation cycle HOIL1 cleavage transiently reduces linear ubiquitination, including of NEMO and RIP1, dampening NF-κB activation and preventing reactivation. By regulating linear ubiquitination, MALT1 is both a positive and negative pleiotropic regulator of the human canonical NF-κB pathway—first promoting activation via the CBM—then triggering HOIL1-dependent negative-feedback termination, preventing reactivation.

## Abstract (web summary)

MALT1 mediates NFκB activation. Here the authors perform proteomic analysis of human immunodeficient mutant MALT1 B cells revealing that MALT1 cleaves the HOIL1 subunit of the linear ubiquitin chain assembly complex to dampen late NFκB activation and to invoke negative feedback of NFκB activation.

## Graphical abstract

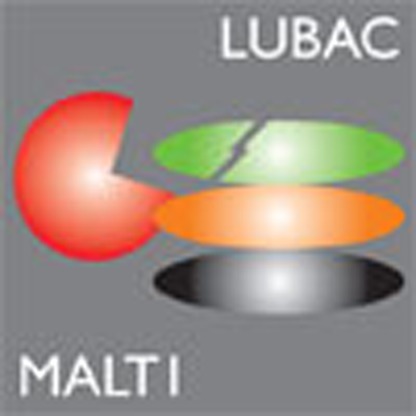

Linear ubiquitin chains, assembled by peptide bond linkage of the ubiquitin Met1 α-amine to the C-terminal glycine of a proximal ubiquitin, are a recently recognized topographic form of polyubiquitination. This modification is highly associated with anti-inflammatory responses^1^, nuclear factor-kappa B (NF-κB) activation and protection from tumour necrosis factor receptor superfamily-mediated apoptosis^2^. Linear ubiquitination E3 ligase activity uniquely resides in heme-oxidized IRP2 ubiquitin ligase (HOIL1)-interacting protein (HOIP). Full HOIP activity requires HOIL1 (refs 3, 4) and Shank-associated RH domain interactor (SHARPIN)^5^,^6^ to activate and stabilize HOIP to form the linear ubiquitin chain assembly complex (LUBAC)^7^,^8^. The linear chain deubiquitinase OTULIN also reversibly associates with HOIP^9^,^10^. Tumour necrosis factor-, CD40L- and IL-1β-induced canonical NF-κB activation requires specific, high-affinity binding of NF-κB essential modulator (NEMO) to proteins modified by linear ubiquitin at cell membrane-anchored receptor signalosomes^1^,^11^,^12^,^13^. Although the importance of LUBAC for NF-κB signalling is highlighted by germline and somatic mutations in LUBAC genes resulting in primary immunodeficiency diseases or in lymphomagenesis driven by NF-κB (refs 14, 15, 16), HOIP catalytic activity can be dispensable for B-cell receptor signalling^17^. Thus, regulation of LUBAC assembly, activity and inactivation remains ill defined.

As a central regulator of innate and adaptive immunity, the NF-κB pathway integrates signals converging from a range of cell surface and intracellular pattern recognition receptors, leading to rapid nuclear translocation of the transcription factor NF-κB (ref. 18). A key convergence point in the NF-κB pathway is the CARD11/BCL10/MALT1 (CBM) signalosome, which consists of the caspase recruitment domain-containing protein 11 (CARD11), B-cell lymphoma/leukaemia 10 (BCL10) and a cysteine protease, mucosa-associated lymphoid tissue lymphoma translocation protein 1 (MALT1)—the only human paracaspase^19^. The CBM signalosome rapidly transduces receptor engagement to the canonical IκB kinase (IKK) complex, consisting of IKKα, IKKβ and IKKγ/NEMO subunits. Linear ubiquitination of NEMO is required for phosphorylation of IκBα by the IKK complex^11^. Phospho-IκBα is then rapidly Lys48-polyubiquitinated, initiating proteasomal degradation and allowing free NF-κB to translocate to the nucleus. Here it transcribes a tightly controlled program of proinflammatory genes and negative regulators of apoptosis (Fig. 1a). The importance of the CBM in immunity is revealed by the profound disruption in T- and B-cell receptor signalling in human and mouse genetic deficiencies for all the CBM components^19^,^20^,^21^,^22^,^23^,^24^,^25^.

In addition to mediating essential protein scaffolding functions required for transducing NF-κB signalling, MALT1 indirectly enhances NF-κB signalling and cell responses by cleaving and inactivating a limited repertoire of proteins that downregulate the canonical NF-κB pathway including the NF-κB subunit RelB^26^ and two deubiquitinases, A20 (ref. 27) and cylindromatosis protein (CYLD), which also cleaves linear ubiquitin^28^. In T-cell receptor responses MALT1 autoproteolytic cleavage promotes NF-κB signalling^29^, whereas cleavage of BCL10 regulates T-cell adhesion^30^. The cleavage of two messenger RNA-binding proteins, the RNAse Regnase-1 (ref. 31) and Roquin^32^, stabilizes T-cell receptor-induced messenger RNAs. Although B-cell regulation by MALT1 is less understood, the functionality of the CBM executed by MALT1 proteolysis is evident in lymphomas^33^,^34^. Activated B-cell-type diffuse large B-cell lymphoma carrying activating oncogenic mutations in CARD11 or in the CD79a/b components of the B-cell receptor are associated with constitutive CBM preassembly and cleavage of MALT1 substrates, and chronic active B-cell receptor signalling^35^. MALT1 overexpression following gene amplification in splenic marginal zone lymphomas^36^ or as a fusion protein with cellular inhibitor of apoptosis-2 (cIAP2) in extranodal marginal zone B-cell lymphomas^35^ further highlights MALT1 as an important drug target for lymphoma^37^.

Recently, we described the only known living patient in the world with a genetic MALT1 deficiency^38^. This now 17-year-old girl presented with combined immunodeficiency associated with immune dysregulation caused by a homozygous loss-of-function mutation in *MALT1*. Heterozygous family members are healthy with no immunological disorders. The MALT1 mutation Trp580Ser breaks a key stabilizing contact of the MALT1 immunoglobulin and catalytic domains, resulting in lower levels of a less active and less stable MALT1-Trp580Ser mutant protein causing failure to activate NF-κB in the patient's T cells^38^. Paradoxically, the patient displays constitutive autoinflammatory responses, particularly affecting her skin and gastrointestinal tract; indeed, she experienced clinical benefit using anti-inflammatory corticosteroids^38^, highlighting the gaps in our understanding of the role of MALT1 in human immunity. Access to human cells deficient in MALT1 provides a unique opportunity to identify new MALT1 substrates in an intact human system without experimental over- or under-expression. Using 10-plex Tandem Mass Tags (TMTs), we identify HOIL1 as a MALT1 substrate directly linking MALT1 paracaspase activity for the downregulation of linear ubiquitination and eventual cessation of NF-κB signalling in a negative-feedback mechanism.

## Results

### TAILS identification of HOIL1 as a new MALT1 substrate

**Figure 1:**
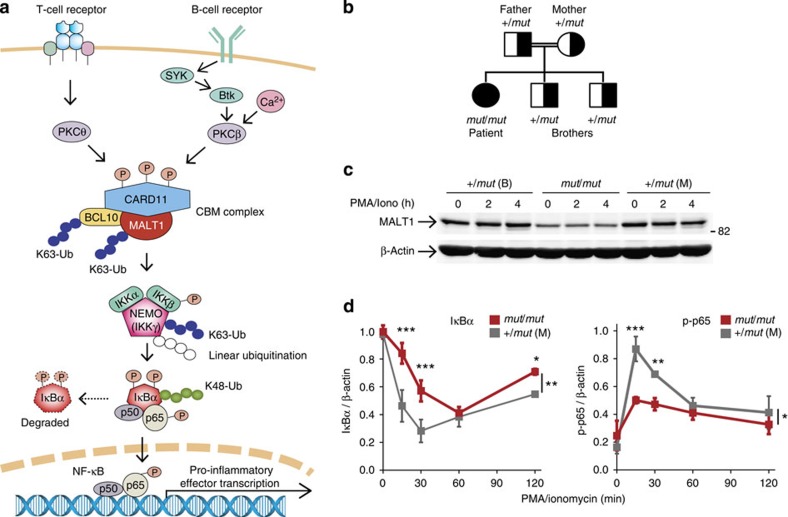
Defective NF-κB activation in *MALT1*^*mut/mut*^ B cells. (**a**) Simplified diagram showing the central role of the CARD11/BCL10/MALT1 (CBM) complex in B- and T-cell receptor controlled canonical NF-κB signalling pathway. (**b**) Family pedigree of the *MALT1* genetic mutation. (**c**) Immunoblots of MALT1 before and after stimulation with PMA/ionomycin for 2 and 4 h in immortalized B cells from the MALT1-(Trp580Ser) homozygous daughter (*mut*/*mut*), the heterozygous brother (+/*mut* B) and mother (+/*mut* M), *N*=11. β-Actin, loading control. (**d**) NF-κB activation deficiency in B cells of patient (*mut*/*mut*) and mother (+/*mut* M) after PMA/ionomycin stimulation was shown by IκBα degradation (left) and phosphorylation of the p65 subunit of NF-κB (p-p65; right), mean±s.d. Bonferroni post-test after two-way analysis of variance: *P<0.05; **P<0.01; ***P<0.001; number of quantified experiments (*N*=3), total numbers of times this was observed (*N*=12).

The search for MALT1 substrates has so far proven intractable by proteomics, in part due to fastidious MALT1 activation mechanisms requiring CBM assembly, and in part from a limited, yet key substrate repertoire that requires high-sensitivity proteomic approaches. MALT1 genetically deficient cells provided a unique opportunity to identify new substrates in an intact human system without experimental over- or under-expression. Immortalized B cells from the patient (*MALT1*^*mut/mut*^; Fig. 1b) had less MALT1 than from her heterozygous (*MALT1*^+/*mut*^) brother and mother, both before and after stimulation by phorbol 12-myristate 13-acetate (PMA)/ionomycin, a strong inducer of NF-κB (*N*=11; Fig. 1c). MALT1-Trp580Ser in *MALT1*^*mut/mut*^ B cells was associated with impaired NF-κB activation as evidenced by delayed and reduced proteasome degradation of IκBα and a >50% loss of activated phospho (p)-p65 (*N*=12 Fig. 1d; Supplementary Fig. 1). Consistent with the genetic pattern of homozygous disease inheritance, MALT1-Trp580Ser in the heterozygous cells did not behave as a dominant-negative protein in NF-κB activation.

**Figure 2:**
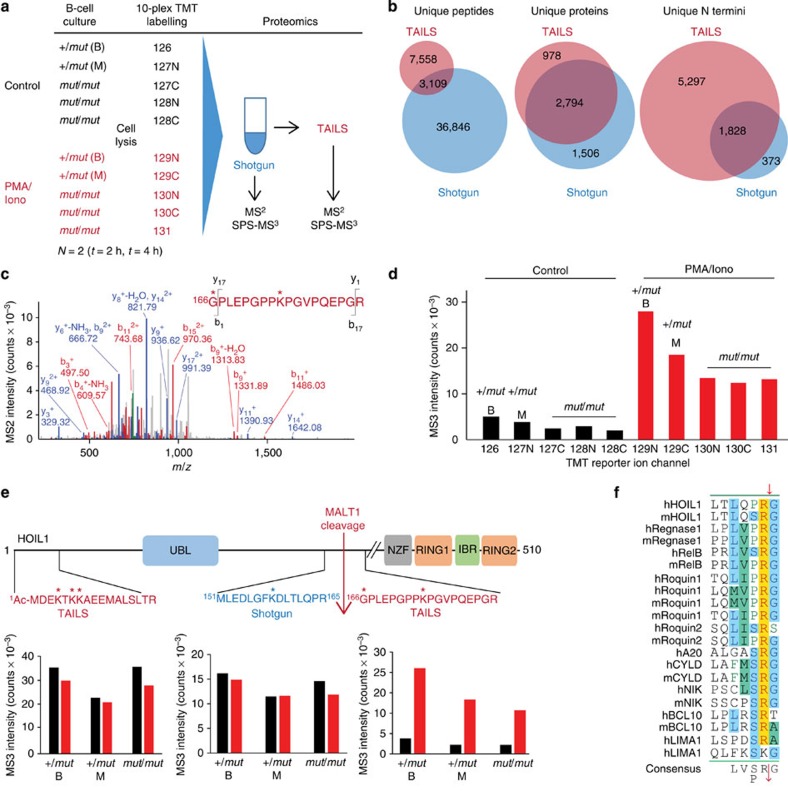
N-terminal TAILS proteomics investigation of patient and control B cells reveal HOIL1 as a MALT1 substrate. (**a**) 10-plex TMT labelling scheme for TAILS and preTAILS shotgun proteomics analyses of immortalized B-cell lysates from the *MALT1* mutant patient (*mut*/*mut*) and heterozygous brother (+/*mut* B) and mother (+/*mut* M) controls after 2 and 4 h stimulation with PMA/ionomycin (PMA/Iono) or solvent (control; *N=2*). Peptides were identified by MS/MS (FDR≤1.0%) and quantified by SPS-MS/MS/MS. (**b**) All unique peptides (including cyclization of glutamine and carry-over of unblocked peptides), unique proteins and unique N-terminal peptides (*N*-acetylated and N-TMT labelled) identified by Byonic in the TAILS and shotgun proteomics analyses at an FDR of 1% at the protein level. Individual peptides were subsequently filtered at a probability >0.99. (**c**) MS/MS spectrum of ^166^GPLEPGPPKPGVPQEPGR^182^, the neo-N-terminal peptide of the carboxy-cleavage product of HOIL1 identified by TAILS. * Represents TMT-labelled residues. (**d**) Absolute intensity values of the 10-plex isobaric reporters from the spectrum in **c** of control (black) and 2 h PMA/ionomycin-stimulated (red) samples. (**e**) MALT1-cleavage site in HOIL1 identified by TAILS from the high reporter ratio of the neo-N-terminal peptide (red) comparing +/*mut* to *mut*/*mut* samples both before (black bars) and after PMA/ionomycin stimulation (red bars; *n*=10). The natural protein N-terminal peptide of HOIL1 was identified by TAILS (red; *n*=3), whereas the nonprime side peptide of the cleavage site was identified by shotgun proteomics (blue; *n*=4). The figure is representative of independent experiments yielding similar results. * Represents TMT-labelled amino acid; Ac, N-terminal acetylation. (**f**) Sequence alignment of the MALT1-cleavage site in HOIL1 with all known MALT1 substrates; m, murine; h, human. Amino acids in all sequences that are identical (yellow), similar (green) and identical in some (blue) are shown.

To identify new MALT1 substrates, we used terminal amine isotopic labelling of substrates (TAILS), a highly sensitive proteomics approach that enriches and identifies protease substrates simultaneously with their cleavage sites by tandem mass spectrometry (MS/MS)^39^,^40^. Isobaric 10-plex TMT labelling blocked protein natural and cleaved neo-N-terminal α-amines in lysate protein of resting and PMA/ionomycin-activated B cells. Following trypsin digestion, we simultaneously compared the TAILS-enriched B-cell N-terminomes from the patient (*n*=3) with her mother (*n*=1) and brother (*n*=1) as controls, without or with PMA/ionomycin stimulation for 2 and 4 h (Fig. 2a) to increase cleaved substrate accumulation for detection. Enabled by the 10-plex TMT-labelling strategy, this was performed in a single mass spectrometric analysis to reduce experimental variability. We also quantified protein changes in the B-cell proteome by shotgun proteomics (that is, preTAILS analysis). We identified 7,498 unique acetylated or TMT-labelled N-terminal peptides (peptide false discovery rate (FDR)≤1%) in 3,772 proteins (protein FDR≤1%). Even before removing decoy peptides and false identifications (*n*=29), the data set had ≤1 reverse hit per 1,000 peptide spectrum matches (Fig. 2b).

To identify high-confidence MALT1 substrates, we analysed TMT-labelled cleaved neo-N-termini by high-accuracy TMT reporter ion ratios using synchronous precursor selection (SPS)-MS/MS/MS (Supplementary Fig. 2) to reduce peak interferences^41^. Substrate winnowing (Supplementary Fig. 3) identified a MALT1-cleavage site at ^165^Arg↓Gly^166^ in HOIL1. The neo-N-terminal peptide ^166^GPLEPGPPKPGVPQEPGR^182^ identifying the C-terminal HOIL1-cleavage product was reproducibly identified in multiple peptide spectrum matches (*n*=10) in every experiment (Fig. 2c). Importantly, it was markedly increased after PMA/ionomycin stimulation and was always present in lower amounts in the *MALT*^*mut/mut*^ cells compared with the *MALT1*^+/*mut*^ cells from both the brother and the mother (Fig. 2d,e; Supplementary Fig. 4a). Finally, this cleavage site complies with the consensus site LXP/SR↓G of the known MALT1 substrates (Fig. 2f). The abundance of the HOIL1 natural N terminus (*n*=5) from the TAILS data and a tryptic peptide lying immediately N terminal to the cleavage site from the preTAILS shotgun data (*n*=3; Supplementary Fig. 4b,c) was constant in all samples confirming that the increase in HOIL1 neo-N-terminal peptide was not merely due to differences in protein abundance after stimulation (Fig. 2e). Hence, with high-confidence TAILS identified HOIL1 as a new MALT1 substrate with ^162^LQPR↓G^166^ as the cleavage site.

Previously described substrates of MALT1 were not identified. This may be explained by cell type specificity of certain protease-substrate interactions as we analyzed B cells, whereas Roquin and Regnase-1 are described in literature as substrates in T cell-specific processes, and LIMA1 and NF-κB-induced kinase (NIK) are lymphoma-enriched substrates. An alternative explanation is that several known substrates yield neo-N-termini unfavourable to mass spectrometry—either they are too small (BCL10: TVSR) or too large (for example, A20: GEAYEPLAWNPEESTGGPHSAPPTAPSPFLFSETTAMKCR; RelB: GAASLSTVTLGPVAPPATPPPWGCPLGR; and CYLD:GVGDKGSSSHNKPKATGSTSDPGNR). Furthermore, posttranslational modifications such as phosphorylation in the neo-N-terminal peptide region may cause mass shifts that render the peptides elusive to identification.

### MALT1 cleaves HOIL1 *in vitro* and in cells

**Figure 3:**
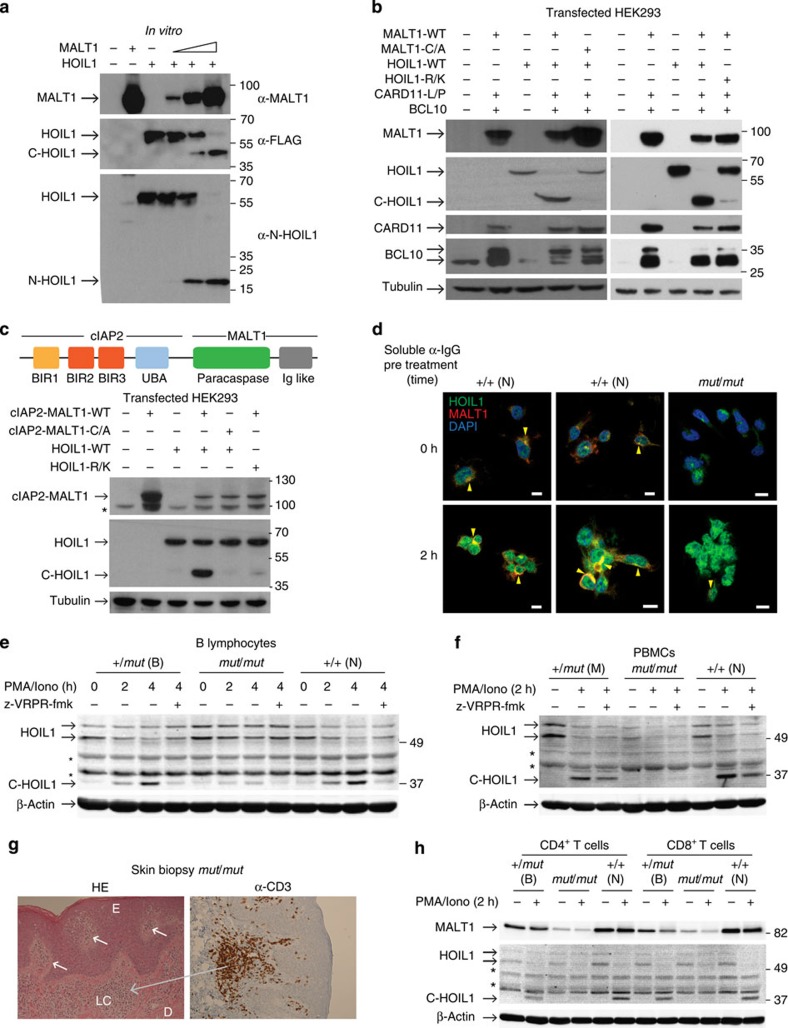
Validation of HOIL1 as a MALT1 substrate *in vitro* and in cells. (**a**) Concentration-dependent *in vitro* cleavage of recombinant human HOIL1 by recombinant human MALT1. Cleavage products were identified using anti-C-terminal FLAG and anti-HOIL1 N-terminal antibodies, *N*=2. (**b**) Immunoblot for HOIL1 cleavage after co-transfection of HOIL1 with CBM proteins: oncogenic mutant CARD11-(Leu244Pro)(L/P), BCL10 and MALT1-(FLAG-His_6_), or catalytically inactive mutant MALT1-(Cys464Ala)(C/A) in HEK293FT cells (left panel), and effect of substitution by charge conserving cleavage site mutation HOIL1-(R165K) (right panel). (**c**) αV5 immunoblot for HOIL1-V5 and cleavage site mutated HOIL1-(Arg165Lys)(R/K)-V5 cleavage by lymphoma fusion protein cIAP2-MALT1-(WT) or the catalytically inactive cIAP2-MALT1-(Cys464Ala)(C/A) in HEK293FT cells. (**d**) Cell–cell contact aligned merged confocal microscopy image slices through the middle of B cells from a normal donor (+/+ N) and the patient (*mut*/*mut*) with and without pretreatment with soluble α-IgG prior to immobilization on α-IgG/IgM coated coverslips and staining. Monoclonal antibody-labelled HOIL1 (green) and MALT1 (red) are shown, yellow arrows indicate co-localization. Blue channel, 4,6-diamidino-2-phenylindole (DAPI) nuclear staining. Scale bar, 10 μm. Individual laser channels for three fields are in Supplementary Fig. 6. (**e**) α-HOIL1 immunoblot for cleavage of endogenous HOIL1 in immortalized B cells from a normal donor (+/+ N) and brother (+/*mut* B) after PMA/ionomycin stimulation with and without preincubation with MALT1 inhibitor z-VRPR-fmk. *N*=34. (**f**) α−HOIL1 immunoblot for HOIL1 cleavage in PMA/ionomycin-stimulated primary peripheral blood mononuclear cells (PBMCs) from the mother (+/*mut* M), a normal donor (+/+N) and the patient (*mut*/*mut*) with and without preincubation with MALT1 inhibitor z-VRPR-fmk. *N*=9. (**g**) Patient (*MALT1*^*mut/mut*^) skin haematoxylin and eosin staining of lymphocytic (LC) infiltrates (white arrows) in the upper dermis (D) surrounding vessels, and the basal layer of the overlying epidermis (E). Immunohistochemistry identified the lymphocytes as CD3^+^ T cells. (**h**) α-HOIL1 immunoblot of HOIL1 cleavage in PMA/ionomycin-stimulated primary CD4^+^ and CD8^+^ T cells from the mother (+/*mut* M), a normal donor (+/+ N) and the patient (*mut*/*mut*), *N*=6. * Represents consistently observed nonspecific band. Tubulin, β-actin: loading controls.

*In vitro* assays in kosmotropic salts^42^,^43^ confirmed HOIL1 as a MALT1 substrate (Fig. 3a). A concentration-dependent single cleavage of C-terminal FLAG-tagged HOIL1 (57 kDa) yielded products of the molecular weight predicted by TAILS—a C-terminal 39-kDa (C-HOIL1) domain and an N-terminal 18-kDa domain (N-HOIL1). To validate cleavage in a cellular context, we established a CBM transfection system in which an active CBM signalosome was assembled in HEK293FT cells in the absence of receptor stimulation by coexpressing MALT1 with BCL10 and the active oncogenic Leu244Pro mutant of CARD11. Cleavage of known substrates A20, CYLD, BCL10 and RelB (Supplementary Fig. 5a–d) co-transfected with the CBM confirmed MALT1-dependent CBM activity. Complete HOIL1 cleavage by the CBM was shown by coexpressing HOIL1 (Fig. 3b). Cleavage was not due to excess levels of MALT1 as HOIL1 was also cleaved by endogenous MALT1 in HEK293FT cells, where endogenous BCL10 was sufficient to form an active CBM with transfected CARD11-Leu244Pro (Supplementary Fig. 5e). HOIL1 cleavage was dependent on MALT1 paracaspase activity—it was not cleaved by the catalytic inactive MALT1-Cys464Ala mutant (Fig. 3b, left). A second Arg-Gly bond in HOIL1 (^180^EPGR-G^184^) lacking the nonprime consensus sequence was not cleaved. Cleavage only occurred at ^165^Arg↓Gly^166^, as a charge conserving HOIL1-Arg165Lys mutant was noncleavable (Fig. 3b, right). The very minor amount of C-HOIL1 generated from mutant HOIL1-Arg165Lys is consistent with weak cleavage at a Lys↓Gly site in LIMA1 (ref. 44; Fig. 2f). Thus, HOIL1 cleavage is site specific with no redundant cleavage by other proteases.

The t(11;18)(q21;q21) chromosomal translocation is the most common translocation found in B-cell lymphomas arising from mucosa-associated lymphoid tissue. This translocation creates the fusion protein cIAP2-MALT1 (Fig. 3c), which exhibits MALT1 proteolytic activity without requiring CBM assembly^35^. Constitutively, active cIAP2-MALT1 fusion protein leads to the cleavage of NIK^45^ and LIMA1 (ref. 44), and acts as an oncogenic driver. Transfected cIAP2-MALT1 cleaved HOIL1 in the absence of CARD11. No cleavage resulted with catalytically inactive cIAP2-MALT1-Cys464Ala mutant or noncleavable HOIL1-Arg165Lys (Fig. 3c), revealing that cIAP2-MALT1-mediated HOIL1 cleavage occurs without the need for CBM assembly.

### Reduced HOIL1 cleavage in *MALT1* ^*mut/mut*^ B and T cells

The immunological relevance of HOIL1 cleavage by MALT1 was shown in several ways. First, we demonstrated that endogenous MALT1 of the CBM, together with HOIL1 of the LUBAC complex, co-localized at the cell membrane following B-cell receptor engagement by anti-IgG in immortalized B cells from a normal donor compared with the patient cells. After 2-h stimulation, HOIL1 was redistributed to the cell membrane, particularly at B-cell:B-cell contacts as discrete caps (Fig. 3d; Supplementary Fig. 6).

We next verified cleavage of endogenous HOIL1 by endogenous MALT1 after stimulation of B cells from a healthy donor and in the same *MALT1*^+/*mut*^ and *MALT1*^*mut/mut*^ B-cell lysates analysed proteomically in multiple replicates (*N*=34; Fig. 3e). Cleavage was markedly reduced in the *MALT1*^*mut/mut*^ B cells, with commensurate increases in both of the two molecular weight forms of intact HOIL1 (refs 7, 8). Similarly, cleavage was reduced by the MALT1-selective inhibitor Z-Val-Arg-Pro-DL-Arg-fluoromethylketone (z-VRPR-fmk)^30^ (Fig. 3e) and by the small-molecule paracaspase inhibitor, mepazine^46^ (Supplementary Fig. 7a).

We observed HOIL1 cleavage in fresh PBMCs from a healthy donor and the heterozygous mother (*N*=9; Fig. 3f). In contrast, HOIL1 cleavage was undetectable in *MALT1*^*mut/mut*^ PBMCs. Skin biopsies from the patient revealed dense chronic lymphocytic infiltrates in the upper dermis surrounding vessels, with focal extension of the inflammatory infiltrates into the basal layer of the overlying epidermis (Fig. 3g). Immunohistochemistry identified these as CD3^+^ T cells prompting examination of MALT1 activity in T cells. HOIL1 was cleaved after PMA/ionomycin stimulation in primary CD4^+^ and CD8^+^ T cells from a healthy donor and the *MALT1*^+/*mut*^ brother, but not from the patient (*N*=6; Fig. 3h). Thus, cleavage was not an effect of Epstein–Barr virus immortalization and occurred with endogenous levels of protease and substrate in normal B and T cells.

### MALT1 cleavage of HOIL1 disassembles LUBAC

**Figure 4:**
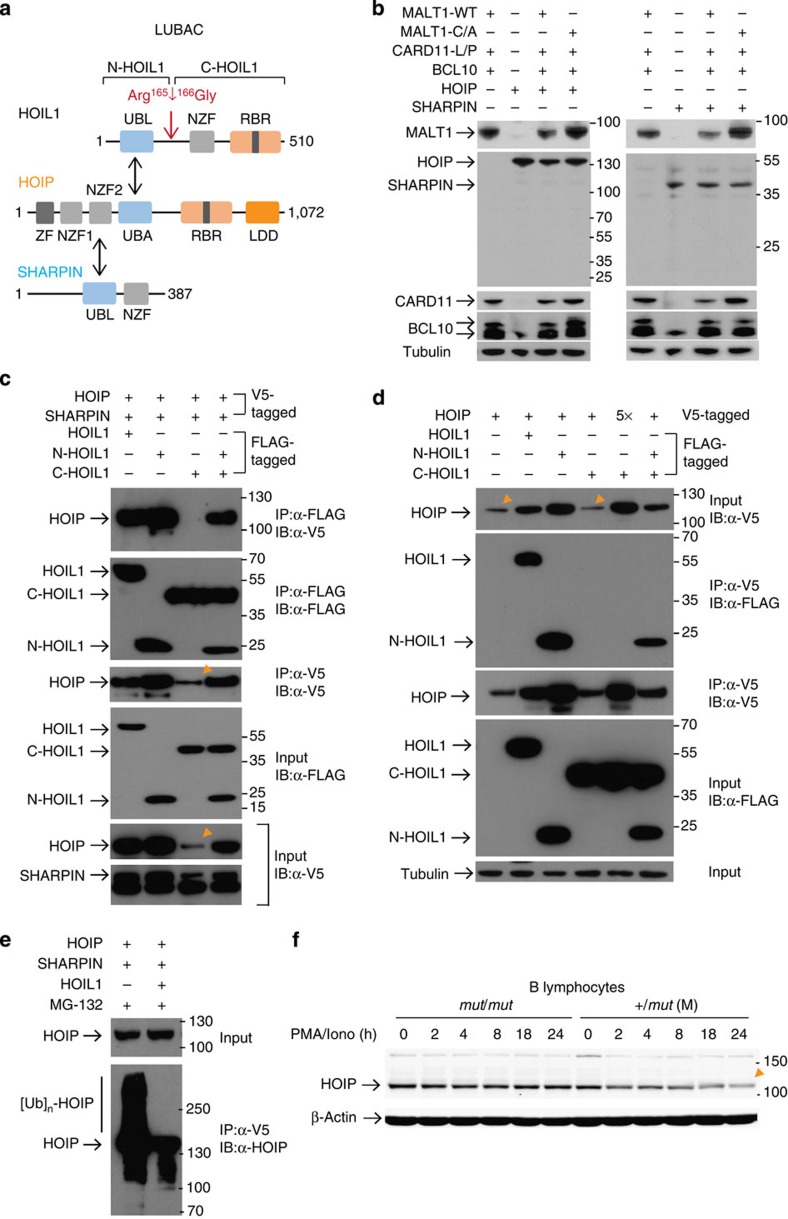
MALT1 remodels LUBAC, releasing C-HOIL1 and reducing HOIP protein level. (**a**) Diagram of LUBAC subunits in relation to the MALT1-cleavage site in HOIL1 that generates N-HOIL1 and C-HOIL1. (**b**) Immunoblots for HOIP and SHARPIN showed MALT1 does not cleave these proteins when active CBM is coexpressed in HEK293 cells. (**c**,**d**) Assembly of LUBAC in HEK293FT cells by co-transfection of HOIL1-FLAG, SHARPIN-V5, HOIP-V5 and five times (5 × ) HOIP-V5 vector. Anti-FLAG (for HOIL1, N-HOIL1 and C-HOIL1) or anti-V5 (for HOIP) immunoprecipitation (IP) and immunoblotting (IB) for HOIL1, N-HOIL1 and C-HOIL1 using anti-FLAG or HOIP using anti-V5 in the immunoprecipitates showed HOIP interaction with HOIL1 and N-HOIL1 but not with C-HOIL1. (**e**) α-V5 IP of lysates of proteasome inhibitor (MG132) treated HEK293FT cells transfected with HOIP-V5, SHARPIN-V5 with or without co-transfection of HOIL1, and subsequent α-HOIP immunoblotting. *N*=3. (**f**) HOIP protein level in B cells from the brother (+/*mut* B) and patient (*mut*/*mut*) on PMA/Ionomycin stimulation over time. β-Actin, loading control, *N*=2. Orange arrowheads indicate decreased HOIP levels.

LUBAC is a complex of HOIL1, HOIP and SHARPIN formed by interaction between the ubiquitin-like, ubiquitin-associated and Npl4 zinc-finger domains of the three LUBAC proteins (Fig. 4a). Neither HOIP nor SHARPIN was cleaved by MALT1 in the cellular context (Fig. 4b). Thus, HOIL1 is the only LUBAC subunit that is a MALT1 substrate.

To mechanistically dissect the effect of MALT1 cleavage of HOIL1 on LUBAC assembly, we coexpressed V5-tagged HOIP and V5-tagged SHARPIN together with FLAG-tagged N-HOIL1 and FLAG-tagged C-HOIL1 cleavage-product analogues (Fig. 4a). Expressing N-HOIL1 with C-HOIL1 represents a non-physiological extreme of complete HOIL1 cleavage, but provides mechanistic insights into the role of HOIL1 in regulating LUBAC assembly and linear ubiquitination. Figure 4c reveals that the N-terminal cleavage product of HOIL1 retains binding interactions with HOIP: V5-tagged HOIP was pulled down by anti-FLAG immunoprecipitation of FLAG-N-HOIL1 (lane 2) to a similar amount as found with full-length FLAG-HOIL1 (lane 1), but not by immunoprecipitation of FLAG-tagged C-HOIL1 (lane 3). N-HOIL1 alone associated with HOIP—as did N-HOIL1 when expressed with C-HOIL1 as an analogue of HOIL1 cleavage (lane 4). Thus, C-HOIL1 neither binds nor modulates binding of N-HOIL1 to HOIP.

We consistently found that HOIP levels were markedly reduced in the absence of HOIL1 (*N=*5; Fig. 4c,d, orange arrowheads)) compared with cells expressing full-length HOIL1 or N-HOIL1 (Fig. 4d). This was not due to unequal transfection efficiency, but shows that N-HOIL1, like full-length HOIL1, stabilizes HOIP. Consistent with this model, HOIP was also reduced when C-HOIL1 alone was expressed with HOIP (*N*=6), suggesting that C-HOIL1 disengages from HOIP on cleavage and does not contribute to HOIP stabilization. However, due to the lower levels of HOIP in the absence of either HOIL1 or N-HOIL1, this is not an equivalent experimental set-up. Therefore, we transfected increasing amounts of HOIP. As shown in Fig. 4d using anti-V5 pulldowns for V5-tagged HOIP, no C-HOIL1 was detected by anti-FLAG immunoblotting, even when HOIP was expressed at equivalent levels using 5 × the transfected amount of HOIP vector. Thus, C-HOIL1 disengages from LUBAC after MALT1 cleavage.

As HOIP is not cleaved by MALT1 (Fig. 4b), we further explored the mechanism of HOIP destabilization in the absence of HOIL1. MG132, a proteasome inhibitor, revealed the presence of polyubiquitinated forms of HOIP when expressed alone (Fig. 4e, lane 1). In contrast, with full-length HOIL1 (Fig. 4e, lane 2), no high-molecular-weight conjugated forms were present. Thus, HOIL1 stabilizes HOIP and protects against proteasome-mediated turnover of HOIP, extending the previous analyses of the stabilizing influence of HOIL1 on HOIP^7^,^47^. Indeed, in *MALT1*^*+/mut*^ B lymphocytes, endogenous HOIP levels showed a gradual and persistent decline up to 24 h after PMA/ionomycin stimulation. This is consistent with the disassembly of LUBAC executed by MALT1 cleavage of HOIL1 with the gradual disengagement of HOIL1 cleavage fragments. In contrast, endogenous HOIP was unaltered over 24 h in the *MALT1*^*mut/mut*^ B cells having reduced HOIL1 cleavage (Fig. 4f). Thus, LUBAC is under dynamic control by MALT1-mediated cleavage of HOIL1. After cleavage, C-HOIL1 releases from LUBAC, whereas N-HOIL1 can maintain its interaction with HOIP.

### HOIL1 cleavage decreases linear ubiquitination by LUBAC

**Figure 5:**
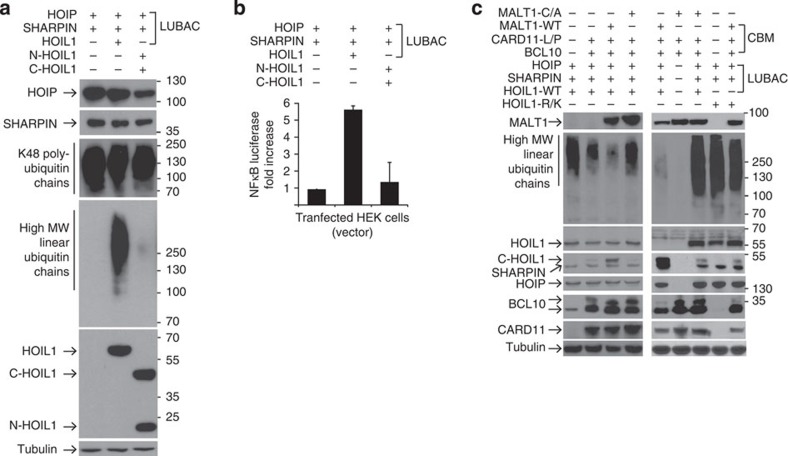
Cleavage of HOIL1 by MALT1 abrogates linear ubiquitination by LUBAC. (**a**) Anti-linear ubiquitin immunoblot to monitor generation of ubiquitinated conjugates on expression of HOIP-V5 and SHARPIN-V5 with full-length HOIL1-V5 or cleavage fragment analogues N- and C-HOIL1-V5 in HEK293FT cells. Total Lys48-polyubiquitination was monitored for reference. Tubulin, loading control, *N*=3. (**b**) Renilla luciferase NF-κB reporter gene assay in the same condition as **a** for transcriptional activity. *N*=2, *n*=3. Data represent mean±s.d. (**c**) Assembly of the LUBAC with CBM complexes by co-transfection of FLAG-tagged oncogenic active mutant CARD11-L/P, BCL10 and MALT1. Anti-linear ubiquitin immunoblotting was performed to monitor generation of high-molecular-weight conjugates in HEK293FT cells transfected with normal LUBAC or LUBAC with noncleavable HOIL1-(R165K), co-transfected with normal CBM or CBM with catalytically inactive MALT1-(C464A). Tubulin, loading control, *N*=2. MW, molecular weight.

LUBAC polymerizes linear ubiquitin chains on immune protein targets. To directly assess the effects of HOIL1 cleavage on linear ubiquitination, we coexpressed HOIP and SHARPIN with either HOIL1 or the N-HOIL1 and C-HOIL1 cleavage analogues in the absence of MALT1. Immunoblotting for linear ubiquitin chains revealed that coexpression of all three intact LUBAC subunits was necessary to efficiently generate high-molecular-weight linear ubiquitin conjugates (Fig. 5a, lane 2), which were virtually abolished by the combination of N-HOIL1 and C-HOIL1 as an analogue of fully cleaved HOIL1 (Fig. 5a). The small amount of linear ubiquitin conjugates is consistent with the less efficient function of LUBAC observed when HOIP is expressed with SHARPIN alone^1^,^5^. The NZF domain in the C terminus of HOIL1 binds linear polyubiquitin^8^ and therefore positions the nascent linear ubiquitin chains proximal to the HOIP E3 ligase. With cleavage the NZF domain in C-HOIL1 is detached from LUBAC reducing LUBAC efficiency. Notably, Lys48-polyubiquitination of proteins was unaffected by the stable HOIL1 cleavage fragments, emphasizing the selectivity in downregulation of linear ubiquitination only (Fig. 5a).

To study the end point of HOIL1 cleavage-induced changes in linear ubiquitination on NF-κB signalling, we quantified NF-κB signalling in the presence and absence of linear ubiquitination using a NF-κB promoter luciferase reporter assay. We found that intact LUBAC with the associated linear ubiquitination of cellular proteins stimulated NF-κB transcriptional activity (Fig. 5b). In contrast, when N-HOIL1 and C-HOIL1 were equally coexpressed as the analogue of fully cleaved HOIL1, along with HOIP and SHARPIN, NF-κB promoter activation was lost, coincident with the reduction in linear ubiquitination (Fig. 5a). Thus, intact HOIL1 is required for efficient LUBAC-dependent linear ubiquitination that is needed for NF-κB promoter activation. Upon HOIL1 cleavage LUBAC is disassembled. This leads to a cessation of linear ubiquitination that results in downregulation of NF-κB activation.

We next expressed both LUBAC and active CBM to determine the effect of MALT1-mediated cleavage of HOIL1 on LUBAC. Consistent with our previous transfection results, we found that when both the CBM and LUBAC were present, total linear ubiquitination was markedly reduced by HOIL1 cleavage (Fig. 5c, lanes 3 and 5). In contrast, LUBAC activity was maintained when cells were transfected with catalytically inactive MALT1-Cys464Ala (Fig. 5c, lanes 4 and 7). Similarly, when the cells were transfected with noncleavable HOIL1-Arg165Lys, linear ubiquitin levels were maintained in the presence or absence of active CBM (Fig. 5c, lanes 8 and 9). Thus, although HOIP is the linear ubiquitination E3 ligase, we found that the CBM reduces HOIP/HOIL1-dependent linear ubiquitination and NF-κB promoter activation that is executed by MALT1 cleavage of HOIL1.

### MALT1 activity reduces linear ubiquitination in B cells

**Figure 6:**
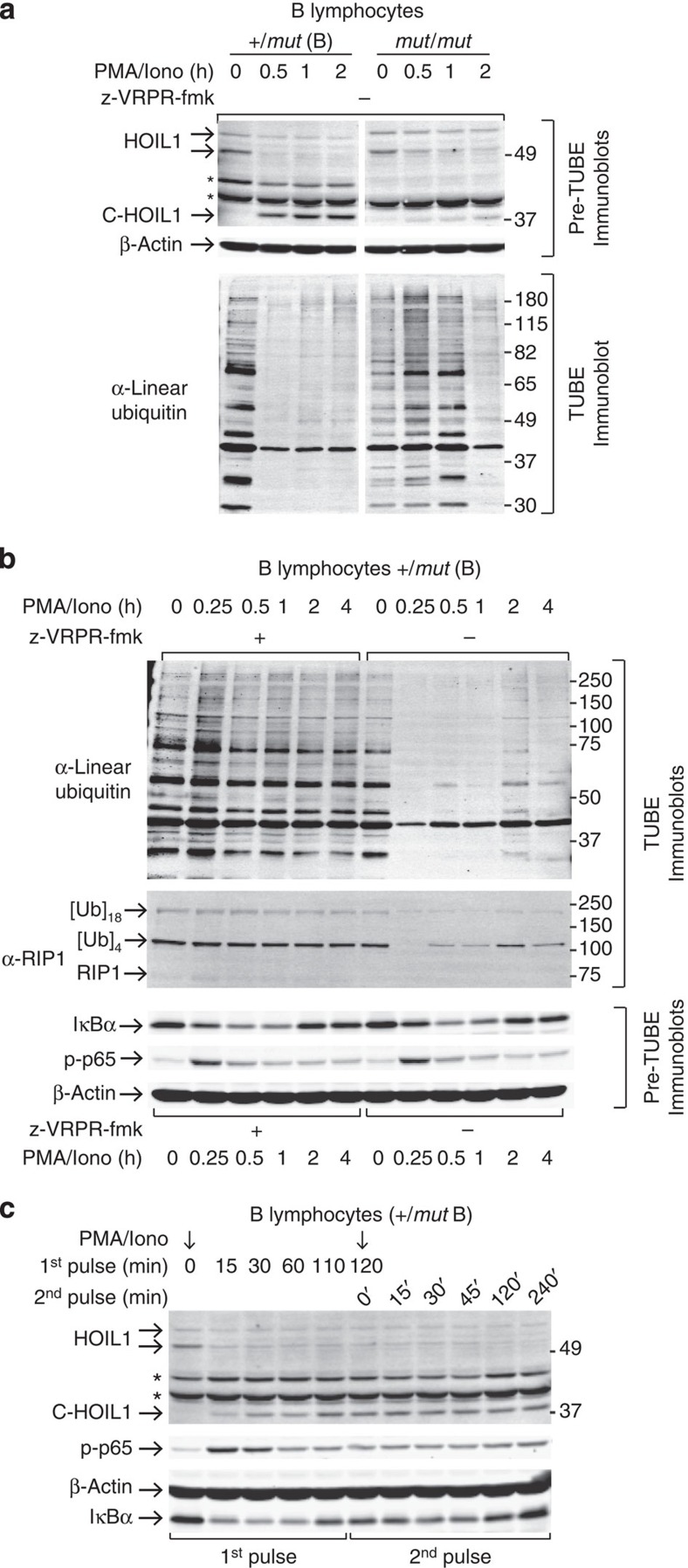
MALT1 proteolysis of HOIL1 in human immortalized B cells reduces linear ubiquitination. (**a**) Immunoblot (lower gel) for linear ubiquitin conjugates in TUBE-enriched total ubiquitinated protein fraction from B cells from the brother (+/*mut* B) and patient (*mut*/*mut*) after stimulation. Generation of C-HOIL1 by MALT1 was shown by α-HOIL1 immunoblot for reference (Pre-TUBE, upper gel). *N*=4. β-Actin, loading control. (**b**) Immunoblot for linear ubiquitin on the TUBE-enriched ubiquitinated protein fraction in B cells from the brother (+/*mut* B) after PMA/ionomycin stimulation, with and without preincubation with MALT1 inhibitor z-VRPR-fmk. *N*=2. Immunoblotting for RIP1 a known substrate of LUBAC, was performed on the same samples. The number of ubiquitin moieties attached to each RIP1-polyubiquitinated species detected was calculated from their apparent molecular weights, and is indicated. NF-κB signalling was monitored by immunoblotting for IκBα and phospho-p65 (p-p65), *N*=6. β-Actin, loading control. (**c**) After initial stimulation by PMA/ionomycin for 15 min followed by cell washing, +/*mut* B cells were again stimulated after washing at 120 min, designated 2^nd^ pulse 0 min. Cells were collected at the indicated times and lysates were probed for phospho-p65 and IκBα levels, *N*=6. β-Actin, loading control on the same blot as IκBα.

To translate these results to lymphocytes, we investigated the impact of HOIL1 cleavage by MALT1 on LUBAC function in B cells. We used agarose beads coupled with tandem ubiquitin binding entities (TUBEs) to enrich for the entire pool of ubiquitinated proteins. Immunoblotting for linear ubiquitin chains in the TUBE-enriched fraction revealed multiple linear ubiquitinated proteins before stimulation in both *MALT1*^+/*mut*^ and *MALT1*^*mut/mut*^ B cells (Fig. 6a, 0 h). Between 30 and 120 min after PMA/ionomycin stimulation in *MALT1*^*+/mut*^ B lymphocytes, there was reduced linear ubiquitination of cellular proteins, which was associated with cleavage of endogenous HOIL1. In the *MALT1*^*mut/mut*^ B cells, both a less pronounced and a delayed reduction in total linear ubiquitination occurred concurrent with greatly reduced cleavage of HOIL1 (*N*=4; Fig. 6a). Importantly, MALT1 inhibition by z-VRPR-fmk reduced HOIL1 cleavage and strikingly maintained total linear ubiquitination levels for up to 4 h in *MALT1*^*+/mut*^ B cells (*N*=6; Fig. 6b) and *MALT1*^*+/mut*^ and *MALT1*^*mut/mut*^ B cells (*N*=6; Supplementary Fig. 7b). Protein levels of OTULIN, the linear polyubiquitin deubiquitinase, were unaltered on PMA/ionomycin stimulation (Supplementary Fig. 7c), reinforcing the direct linkage between MALT1 cleavage of HOIL1 and reduced linear ubiquitination.

RIP1 is a substrate of LUBAC and showed a rapid (<15 min) loss in ubiquitinated forms after stimulation that was then followed by partial recovery in *MALT1*^+/*mut*^ cells (Fig. 6b). This pattern matched the markedly reduced levels of multiple linear ubiquitinated protein conjugates detected by immunoblotting with anti-linear ubiquitin antibody (Fig. 6b). Importantly, MALT1 inhibition abrogated both the general protein and the RIP-specific ubiquitination response to PMA/ionomycin stimulation demonstrating that MALT1 cleavage of HOIL1 reduces linear ubiquitination of both total proteins and the LUBAC target, RIP1.

Intriguingly, the initial triggering of NF-κB signalling did not require MALT1 paracaspase activity, as shown by the similar initial reduction in IκBα and induction of p-p65 both with and without inhibitor in both *MALT1*^+/*mut*^ and *MALT1*^*mut*/*mut*^ B cells (*N=*6; Fig. 6b; Supplementary Fig. 7b). This suggests that the scaffolding role of MALT1 is more important than its proteolytic activity for the initiation of NF-κB signalling. In contrast, cleavage of HOIL1 by MALT1 decreases linear ubiquitination of multiple proteins at later time points. Because RIP1 and NEMO promote NF-κB activation in a manner that depends on their linear ubiquitination, we posited that MALT1-dependent cleavage of HOIL1 may limit continued NF-κB activation. Following an initial 15-min stimulation with PMA/ionomycin, we re-stimulated again at 2 h, a time point when linear ubiquitination was greatly diminished. We then followed cell responses until 4 h (Fig. 6c). Notably, the p-p65 levels did not increase after the second stimulus indicative of no further NF-κB induction (Fig. 6c). Although only correlative, these data are consistent with the relative balance in levels of intact and MALT1-cleaved HOIL1 modulating the degree of linear ubiquitination and hence degree of NF-κB activation. Notably, when no linear ubiquitinated protein was present, cells were rendered refractory to reactivation.

### Stimulation of primary lymphocytes reduces linear ubiquitin

**Figure 7:**
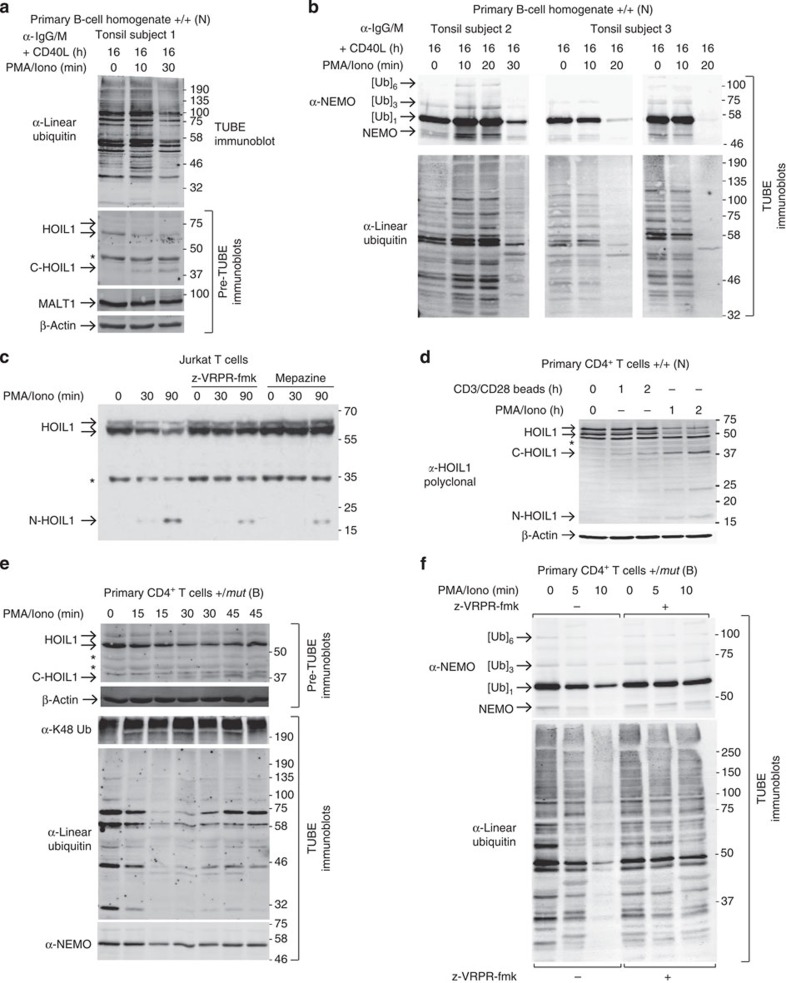
Cleavage of HOIL1 by MALT1 leads to decreased linear ubiquitination in primary lymphocytes. (**a**) Immunoblotting for total linear ubiquitination and HOIL1 cleavage in the TUBE-enriched fraction from α-IgG/M-CD40L primed tonsil B-cell homogenates from a normal (+/+N) subject on PMA/ionomycin stimulation. * Represents nonspecific band. β-Actin, loading control. (**b**) TUBE pulldown and subsequent immunoblotting for linear ubiquitin conjugates and the known LUBAC target NEMO in tonsil B-cell homogenates from normal (+/+N) subjects on stimulation with PMA/ionomycin. Cells were primed with α-IgG/M and CD40L. NEMO-polyubiquitin conjugates were calculated as marked. *N*=7 for **a** and **b**. (**c**) α-N-HOIL1 immunoblot for PMA/ionomycin-induced cleavage of HOIL1 in Jurkat T cells with and without MALT1 inhibitors z-VRPR-fmk (20 μM) and Mepazine (20 μM). * Represents nonspecific bands. (**d**) α-HOIL1 immunoblot for HOIL1 cleavage on stimulation of expanded primary CD4^+^ T cells from the brother (+/*mut* B) with PMA/ionomycin and with α-CD3/α-CD28 coated beads (*N*=3). The polyclonal antibody detects both the C-terminal and the N-terminal cleavage products. * Represents consistently observed nonspecific band. β-Actin, loading control. (**e**) Immunoblots for total linear ubiquitination after TUBE pulldown in expanded primary CD4^+^ T cells from the brother (+/*mut* B) on stimulation with PMA/ionomycin. K48-linked ubiquitination was included for reference. β-Actin, loading control. (**f**) Immunoblots for total linear ubiquitin conjugates and NEMO in PMA/ionomycin-stimulated expanded primary CD4^+^ T cells from the brother (+/*mut* B), with and without pretreatment with MALT1 inhibitor z-VRPR-fmk (*N*=3). NEMO-polyubiquitin conjugates were calculated as marked.

To confirm that the loss of linear ubiquitination after cleavage of HOIL1 is physiologically relevant, we analysed primary human lymphocytes. First, we obtained B-cell-rich cell homogenates from resected paediatric human tonsils. Cells were stimulated first with anti-IgG/IgM and CD40 ligand, and then with PMA/ionomycin. HOIL1 cleavage (Fig. 7a) was associated with a clear decrease in linear ubiquitination 20–30 min after PMA/ionomycin stimulation in multiple human samples (*N=*4; Fig. 7a,b). Moreover, the abundance of several distinct ubiquitinated forms of NEMO was also decreased coinciding with the loss of total linear ubiquitination (Fig. 7b).

Since obtaining sufficient numbers of blood-derived primary B cells for TUBE experiments from the patient was not ethically possible, we further validated the effect of HOIL1 cleavage on linear ubiquitination by returning to T cells, which skin biopsy had shown were dominant in the upper dermis (Fig. 3g). On PMA/ionomycin stimulation of Jurkat T cells, HOIL1 levels were reduced; and the role of MALT1 cleavage was confirmed by the reduced generation of N-HOIL1 with the MALT1 inhibitors z-VRPR-fmk and Mepazine (Fig. 7c).

To show that cleavage of HOIL1 by MALT1 in T cells is a downstream consequence of lymphocyte antigen receptor/co-stimulatory receptor engagement, we incubated primary CD4^+^ T cells from healthy donors (*N*=3) with anti-CD3/anti-CD28 functionalized beads. T-cell receptor/CD28 engagement stimulated HOIL1 cleavage (Fig. 7d), but to a lesser extent than the pharmacological PMA/ionomycin stimulation. Thus, HOIL1 cleavage is a consequence of B- and T-cell stimulation with PMA/ionomycin and of T-cell receptor engagement.

Next, primary CD4^+^ T cells from the heterozygous brother were stimulated with PMA/ionomycin. As with B cells, we observed a time-dependent decrease in linear ubiquitination (Fig. 7e) and specifically of NEMO (Fig. 7f). In contrast, total Lys48-polyubiquitination remained constant on stimulation, confirming the specificity of the reduction to linear ubiquitination (Fig. 7e). The loss of linear ubiquitin conjugates, and specifically of NEMO ubiquitination, was blocked by MALT1 inhibition (Fig. 7f), emphasizing that this effect in human T cells was dependent on MALT1 paracaspase activity and cleavage of HOIL1.

**Figure 8:**
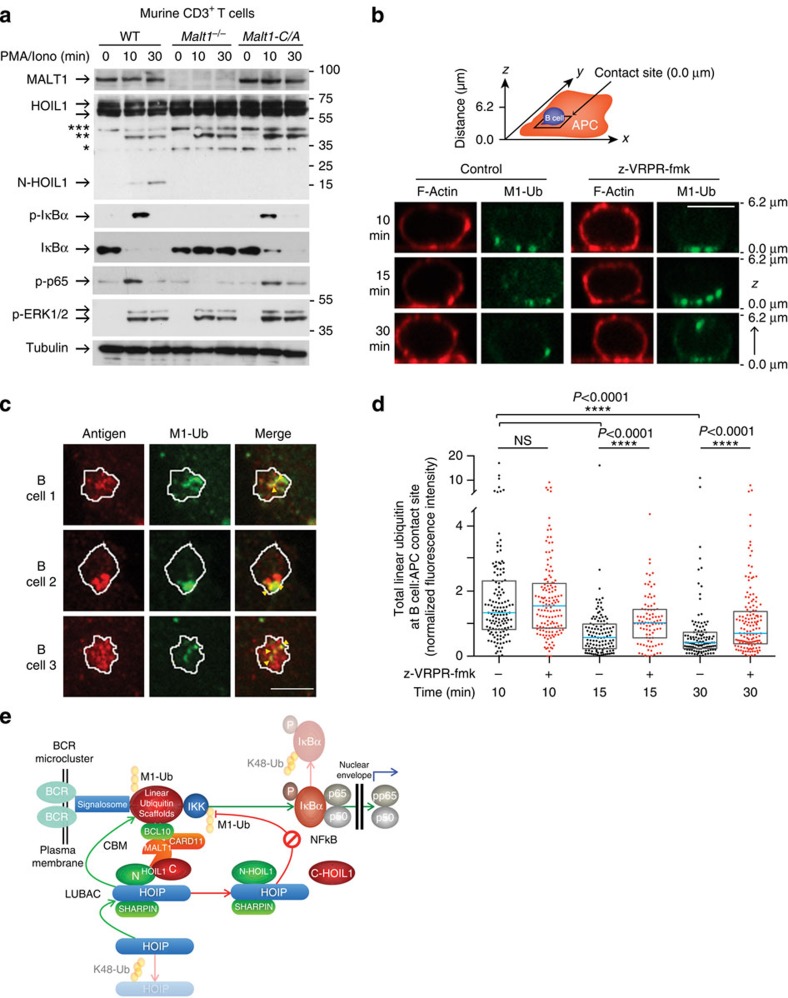
MALT1 cleavage of HOIL1 decreases linear ubiquitination in mouse lymphocytes. (**a**) Immunoblots for N-HOIL1, p-ERK1/2 and NF-κB signalling proteins (p-IκBα, IκBα and p-p65) in stimulated murine primary lymph node CD3^+^ T cells derived from wild-type mice, the littermate *Malt1*^−/−^ and the *Malt1-C/A* catalytic inactive mutant knock-in to *Malt1*^−/−^, *N*=2. Tubulin, loading control. * Represents nonspecific band as observed in Jurkat T cells; ** Represents residual signal from p-ERK1/2 blot; *** Represents nonspecific bands and residual p-ERK1/2 signal. (**b**) Murine splenic B cells treated with vehicle (control) or 75 μM MALT1 inhibitor (z-VRPR-fmk) for 30 min were exposed to APCs for the indicated times. *z*-Slices through the centre of B cells stained for F-actin (red) and linear polyubiquitin (M1-Ub, green) are shown. Scale bar, 5 μm. (**c**) Representative spinning disk confocal *xy* slices at the contact site of three individual primary murine splenic B cells that were added to APCs for 10 min before staining for the anti-Igκ surrogate antigen (red) and M1-linear polyubiquitin (green). White lines show the B-cell periphery at the contact site, as visualized by F-actin staining. Yellow arrowheads show sites of linear ubiquitin and antigen microcluster co-localization. Scale bar, 5 μm. (**d**) Normalized linear ubiquitin fluorescence intensity in the *xy* plane at the B cell:APC contact site was quantified from images similar to those in **c** and normalized for the amount of antigen staining at each contact surface. Each dot represents a single B cell. Blue line depicts the median value of *n*=91–120 B cells from two independent experiments (*N*=2). Box shows interquartile range. ****P<0.0001 using the Mann–Whitney *U*-test. (**e**) Scheme depicting normal initiation of NF-κB signalling by B-cell receptor (BCR) microclusters (green arrows) via MALT1 scaffolding in the CBM and the canonical IκB kinase (IKK) complex, consisting of NEMO/IKKγ with IKKα and IKKβ subunits. In the signal amplification stage, MALT1 cleavage removes negative regulators and thereby optimizes NF-κB activation. The late-stage negative-feedback loop is executed by MALT1 paracaspase activity cleaving HOIL1 in LUBAC (red arrows) to transiently reduce linear ubiquitination of targets including NEMO/IKKγ, hence reducing NF-κB signalling at late time points and is associated with preventing further ongoing NF-κB stimulation.

To widen the physiological relevance of our findings, we showed that HOIL1 cleavage occurred 30 min after PMA/ionomycin stimulation of primary murine lymph node CD3^+^ T cells, but not in knockout mouse cells lacking MALT1 or in cells from catalytic inactive MALT1-Cys/Ala knock-in mice (Fig. 8a). Therefore, HOIL1 cleavage is not human specific, emphasizing the broader relevance of HOIL1 as a substrate. In wild-type murine CD3^+^ T cells, HOIL1 cleavage occurred 30 min after stimulation. At this later time, and as shown by the cessation of the sudden increase in p-IκBα and p-p65, the initial NF-κB activation at 10 min had ended. We next compared the scaffolding versus paracaspase activities of murine MALT1 in NF-κB activation in PMA/ionomycin-stimulated primary murine lymph node CD3^+^ T cells. Ten minutes after PMA/ionomycin stimulation, both the wild-type and catalytic inactive MALT1-C/A knock-in murine T cells showed similar levels of induced p-p65 and p-IκBα that was coupled with reduced IκBα. In contrast, in the absence of MALT1 in *Malt1*^−/−^ mice, NF-κB was not activated, whereas phosphorylation of ERK1/2 was indistinguishable and confirmed cellular activation (Fig. 8a). Thus, the murine *Malt1* knockout and knock-in studies in T cells confirm the human B-cell MALT1 inhibitor data showing that MALT1 paracaspase activity was dispensable for NF-κB activation.

### B-cell activation by antigen-presenting cells reduces LUBAC

*In vivo*, B cells are often activated by antigen-presenting cells (APCs) that capture antigens to display on their surface^48^. The binding of B-cell receptors to mobile membrane-bound antigens leads to forming B-cell receptor microclusters that nucleate signalosome formation and activate downstream signalling pathways^49^. Because linear ubiquitin chains act as scaffolds to recruit and organize signalling proteins^50^, we asked whether they accumulate at the B cell:APC contact site (that is, immune synapse), and specifically at B-cell receptor microclusters. Primary murine splenic B cells were added to Cos7 APCs that express on their surface a transmembrane form of an anti-Igκ light-chain antibody (that is, surrogate antigen) that binds to all B-cell receptors^51^. B cells do not attach to Cos7 cells not expressing this surrogate antigen. At 10 min after adding B cells to anti-Igκ-expressing Cos7 APCs, puncta of linear ubiquitin staining were observed at the B cell:APC contact site by spinning disc confocal microscopy (Fig. 8b). The punctate linear ubiquitin staining also co-localized with B-cell receptor microclusters, which were visualized as puncta of the surrogate antigen (Fig. 8c). Although the linear ubiquitin puncta co-localized with B-cell receptor microclusters, only a subset of B-cell receptor microclusters was associated with linear ubiquitin. *z*-Sections through the centre of the B cell showed that linear ubiquitin conjugates were found primarily at the B cell:APC contact site, often at sites where antigens were concentrated at B-cell receptor microclusters.

Time courses showed that the accumulation of linear ubiquitin-conjugated proteins at the B cell:APC contact site was significantly lower (*P*<0.0001) at 15 and 30 min after adding the B cells to the APCs than at the 10-min time point (Fig. 8d). Thus, the time course for the decrease in linear ubiquitination in primary murine B cells stimulated through the B-cell receptor matched those for primary human B and T cells (Fig.7). Notably, we found that MALT1 paracaspase activity was important for the time-dependent decrease in linear ubiquitination-modified proteins. Pretreating the B cells with the z-VRPR-fmk inhibitor significantly delayed the loss of total linear ubiquitination at the B cell:APC contact site at the 15 and 30 min time points (*P*<0.0001; Fig. 8d).

## Discussion

Our discovery of a new MALT1 substrate, which was enabled by a unique patient with genetic MALT1 deficiency, has defined a new biological role for MALT1. We report a previously unrecognized yet direct proteolytic connection between the CBM signalosome and LUBAC (Fig. 8e). By transiently reducing linear ubiquitination at the lymphocyte antigen receptor microcluster, MALT1 displays temporally separated actions in NF-κB signalling—initially as a feedforward inducer and then ultimately as a negative-feedback inhibitor. To date, MALT1 proteolytic activity has only been proposed to indirectly increase NF-κB activation by cleavage and removal of negative regulators of the canonical NF-κB pathway. However, we show that MALT1 also results in a single specific cleavage in HOIL1, disrupting LUBAC by release of C-HOIL1, to downregulate linear ubiquitination at the B-cell receptor microcluster signalosome. The NZF domain in C-HOIL1 binds LUBAC to linear ubiquitin chains and so is key for LUBAC E3 ligase efficiency. Therefore, HOIL1 cleavage impairs linear polyubiquitin formation and is associated with reduced LUBAC-mediated NF-κB activation. Loss of MALT1 proteolytic activity in the patient lymphocytes and, with failure to cleave HOIL1, led to an increase in linear ubiquitination levels. This eventually rendered the patient with unrestricted B-cell activation leading to chronic inflammatory responses.

We also observed that triggering of NF-κB signalling did not require MALT1 paracaspase activity. A similar initial reduction in IκBα and induction of p-p65 occurred both with and without a MALT1 inhibitor in activation of human B cells and of murine T cells from wild-type and MALT1-Cys/Ala protease-inactive knock-in mice. This adds a new layer to our understanding of the molecular links in CBM complex and LUBAC crosstalk with NF-κB signalling. Moreover, our results showing first positive and then negative regulation of NF-κB activation by MALT1 indicate that therapeutic intervention with MALT1 inhibitors for autoimmunity or lymphoid neoplasia needs to be considered carefully.

The HOIL1 cleavage site is proximal to Gln185, one of two germline mutation sites in *HOIL1*-*mutant* immunodeficient patients^52^ that have clinical overlap with features of the *MALT1*^*mut/mut*^ patient^38^. Although Nakamura *et al.*^53^ reported HOIL1 cleavage, they did not identify the responsible protease or the physiological consequences. In primary B and T lymphocytes, we found reductions in both total protein linear ubiquitination and levels of the specific target proteins, NEMO and RIP1, on HOIL1 cleavage. This loss of proteins modified by linear ubiquitin was blocked by inhibition of MALT1; thereby directly linking reduced linear ubiquitination with MALT1 paracaspase activity. In view of the indirect amplification of NF-κB by the previously described MALT1-cleavage substrates^27^,^28^,^29^,^30^,^31^,^32^, it was surprising that MALT1 inhibition did not alter p-p65 and IκBα levels on initiation of the NF-κB pathway. This is in agreement with very recent reports also showing the key role of MALT1 scaffolding in NF-κB induction^54^,^55^,^56^. Indeed, initiation of NF-κB signalling in *MALT1*^*mut/mut*^ B cells was dampened compared with healthy *MALT1*^+/*mut*^ family members due to disruption of MALT1 scaffolding caused by low levels of unstable mutant MALT1-Trp580Ser protein.

The possible involvement of LUBAC-mediated linear ubiquitination in B-cell receptor signalling was only recently postulated^16^,^57^. As direct induction of linear ubiquitination has not been previously observed in B-cell receptor signalling and in view of the rearrangement of linear ubiquitin scaffolds to the cell membrane-anchored receptor signalosome that we observed, we posit that ubiquitin scaffolds are essential both proximal and distal to the CBM complex at early time points after B-cell receptor engagement. Indeed, we showed co-localization of CBM and LUBAC at the cell membrane followed by loss of linear ubiquitinated proteins after B-cell receptor engagement. Hence, the cleavage of HOIL1 by MALT1 with the subsequent removal from the signalosome may be another, yet quite different switch by which B cells regulate NF-κB signalling after B-cell receptor activation. This negative-feedback mechanism may serve to avoid undesired processes associated with unrestricted B-cell activation such as in autoimmunity. Indeed, the reduced ability of the *MALT1*^*mut/mut*^ patient to cleave HOIL1 and disassemble LUBAC may explain her excessive chronic inflammatory responses that are a challenging clinical aspect of human MALT1 deficiency^19^,^35^. Through this enhanced understanding of critical interactions between MALT1 and LUBAC, we now have a mechanistic explanation for the patient's seemingly contradictory clinical phenotype—which encompassed both susceptibility to infection and exaggerated inflammation due to persistent canonical NF-κB signalling. Whereas the failure to activate NF-κB quickly and appropriately during microbial challenge resulted in increased susceptibility to infections, the reduced ability to cleave HOIL1 and reduce linear ubiquitination is consistent with unrelenting NF-κB signalling leading to a destructive chronic inflammation of her skin and gastrointestinal tract that was only partially controllable with systemic corticosteroid therapy.

The diagnosis and molecular characterization of rare human primary immunodeficiency diseases plays a critical role in expanding our understanding of the human immune system and in the development of new treatments that have applications beyond immunodeficiency diseases. We used TAILS to directly study the complex consequences of a genetic MALT1 deficiency in humans. This contrasts the usual situation where inferences are drawn by necessity from animal models. HOIL1 is a component of the LUBAC complex, which catalyses linear ubiquitination of several important components and regulators of the canonical NF-κB machinery, including RIP1 and NEMO. By identifying HOIL1 as a novel MALT1 substrate, we bring functional insight to CBM/LUBAC crosstalk. From accumulating data and our present results, we suggest that MALT1 has three temporally separated roles in canonical NF-κB signalling—for the initial triggering of NF-κB, MALT1 protease activity is not required for IKK activation and NF-κB signalling. Rather, MALT1 scaffolding links the CBM signalosome to the B-cell receptor microcluster, which we show is co-localized with linear ubiquitin complexes. In the next time step, MALT1-dependent direct cleavage removes negative regulators such as RelB, CYLD and A20, and thereby optimizing NF-κB activation and cellular responses. At later stages, MALT1 proteolysis invokes an overall negative-feedback mechanism by decreasing LUBAC function, thereby reducing linear ubiquitination that contributes to rendering lymphocytes refractory to undesired ongoing and secondary NF-κB activation (Fig. 8e). Thereby, MALT1 is a pleiotropic controller of canonical NF-κB activation.

## Methods

### Human blood donors and ethics approvals

The University of British Columbia/Children's and Women's Health Centre of British Columbia Research Ethics Board approved the research protocols for studies on human samples and research was conducted in accordance with the Declaration of Helsinki. Five members of the family, including the affected child, her parents and two unaffected siblings, as well as seven healthy volunteers were enrolled. Tonsil samples were deidentified. Written informed consent and assent from minors for participation in this study were obtained.

### Primary hematopoietic cell culture

Human PBMCs were isolated from fresh donor blood samples by Ficoll-Plaque (GE Healthcare Life Sciences) density centrifugation as described earlier^58^. Fresh PBMCs (2 × 10^6^ cells) were cultured overnight in complete RPMI 1640 medium supplemented with 10% fetal bovine serum, 2 mM L-glutamine (all from HyClone Fisher Scientific) and 1 mM sodium pyruvate (Invitrogen). Cells were stimulated with 50 ngml^−1^ PMA and 1 μM ionomycin, with or without 1-h pretreatment with the MALT1 inhibitor z-VRPR-fmk (75 μM, Enzo Life Sciences).

### T-cell isolation

Activated primary CD4^+^ and CD8^+^ T lymphocytes were expanded by the following procedure: fresh PBMCs were stained with fluorescent-conjugated anti-CD4 (RPA-T4) and anti-CD8 (HIT8a) antibodies (both from BD Biosciences) and sorted using a BD FACSAria flow cytometer (BD Biosciences). Purified CD4^+^ and CD8^+^ T cells (2 × 10^5^ cells) were stimulated with phytohaemagglutinin (1 μg, Sigma-Aldrich), IL-2 (100 U, Novartis) and allogeneic feeder PBMCs (1 × 10^6^ cells, irradiated with 5,000 rad, 50:50 mixture from two donors) and JY Epstein–Barr virus (EBV)-immortalized lymphoblastoid B cells (2 × 10^5^ cells, irradiated with 7,500 rad) in 1 ml complete RPMI medium supplemented with 2% blood-type AB human serum, 100 U ml^−1^ penicillin, 100 μg ml^−1^ streptomycin, non-essential amino acids and 5 μM β-mercaptoethanol (all from Life Technologies). The activated CD4^+^ and CD8^+^ T cells were cultured for up to 2 weeks and were provided with fresh RPMI medium supplemented with IL-2 (100 U ml^−1^) every 2–3 days. Expanded T cells (1 × 10^6^ cells) were then cultured overnight with RPMI 1640 medium before PMA/ionomycin stimulation (2 h), with or without 1-h pre-treatment with z-VRPR-fmk.

### T-cell isolation from murine lymph nodes

To prepare primary murine CD3^+^ T cells from 10-week-old male C57Bl/6 (*n*=2), *Malt1*^−/−^ (*n*=2) and *Malt1*-C/A (*n*=2) mice^56^, lymph nodes were isolated and pooled before homogenization using 70-μm cell strainers. Subsequently, erythrocytes were lysed in 0.15 M NH_4_Cl, 10 mM KHCO_3_, 1 mM EDTA and Pan T cells were purified by MACS negative depletion (mouse Pan T-cell Isolation Kit II, Miltenyi Biotec). The purity of the isolated CD3^+^ T cells was confirmed to be >95% by flow cytometry and the T cells were cultured in serum-free RPMI 1640 medium until stimulation. Procedures involving animals were carried out on Experimental Animal Licenses approved by the Novartis Animal Ethics Board and the Regional Governmental Authorities of Switzerland.

### B-cell immortalization

Immortalized B cells were established by standard EBV transformation^59^. In brief, EBV infections were performed by culturing fresh PBMCs from the donors with the supernatant from a viral replication-permissive marmoset cell line B95–8 (VR-1492, ATCC)^60^. After 24-h incubation, cells were infected again with EBV and cultured until sufficient B-cell blasts were present. These were then cultured in complete RPMI 1640 medium (HyClone, Fisher Scientific) for two passages before any experiments. All experiments on EBV-immortalized B cells were performed within 20 passages to avoid potential alteration of the cell phenotype. For immunoblotting analyses, EBV-immortalized B cells (2 × 10^6^ or 4 × 10^6^ cells) were stimulated with PMA/ionomycin for up to 4 h, with or without 1-h pretreatment with z-VRPR-fmk. For the restimulation experiments, *MALT1*^+/*mut*^ cells (2 × 10^6^) were stimulated with PMA/ionomycin for 15 min, and then washed and rested for 2 h before the second PMA/ionomycin stimulation over time (*N=*6). All B- and T-cell cultures were mycoplasma negative.

### Preparation of tonsil cell isolates

The University of British Columbia and the Children's and Women's Health Centre of British Columbia Clinical Review Board (#H06-03256) approved the collection of tonsils from elective surgeries performed at British Columbia Children's Hospital. Subsequently, single-cell suspensions were generated by pressing tissue through filter screens, subjected to Ficoll-Paque (GE Healthcare) density gradient centrifugation and isolated mononuclear cells cryopreserved at −80 °C.

### Statistical analysis

A Bonferroni post-test after two-way analysis of variance was performed for statistical analysis of the difference between response in heterozygous mother and homozygous patient (*N*=3). The data met the assumptions of the test including the dependent variable (that is, protein expression) being continuous, the two independent variables each consisted of five time points, and the time points were independent. There was an estimate of variation within each group of data displayed by the s.d. error bars. The F-test statistic established that variance was similar between the groups that are being statistically compared. Statistical analysis of the effect of MALT1 inhibitor z-VRPR-fmk on linear ubiquitination at the B-cell receptor microclusters was performed by Mann–Whitney *U*–test, since data were not normally distributed and therefore no assumptions about variance were made.

### Confocal immunofluorescence

Immortalized B cells (12.5 × 10^6^ cells per millilitre) in RPMI medium plus 10% cosmic calf serum were used unstimulated or stimulated in suspension for 2 h with 10 μg ml^−1^ anti-IgG (Jackson Immunoresearch Laboratories; 109-005-044) and allowed to settle for 1 h onto coverslips coated with anti-IgG and anti-IgM (Jackson Immunoresearch Laboratories; 109-005-003 and 109-005-043, respectively). After fixation with 4% paraformaldehyde, the cells were permeabilized with 0.5% Triton X-100 in PBS for 5 min and then blocked with 3% bovine serum albumin in PBS for 30 min. Primary antibodies (1:100) were added for 1 h and visualized with Alexa 488- or Alexa 647-labelled goat anti-mouse IgG or goat anti-rabbit IgG. Monoclonal anti-human HOIL1 (clone E-2) and anti-human MALT1 (clone EP603Y) antibodies were from Santa Cruz Biotechnology and Abcam, respectively. Coverslips were treated with ProLong Gold antifade reagent containing 4,6-diamidino-2-phenylindole (Molecular Probes). Images were captured using a Nikon C2+ confocal microscope.

### *In vitro* HOIL1 cleavage assays

Recombinant full-length human MALT1 protein was expressed and purified^43^. C-terminal Myc-FLAG-tagged full-length human HOIL1 was obtained from Origene. HOIL1 (0.05 μg μl^−1^) was incubated with different concentrations of MALT1 in assay buffer (200 mM Tris-HCl, 0.8 M Na-citrate, 0.1 mM EGTA, 0.05% CHAPS, 1 mM DTT, pH 7.4)^43^ for 2 h at 37 °C. Cleavage of HOIL1 was analysed by 4–12% Bis-Tris SDS–polyacrylamide gel electrophoresis (PAGE) gradient gels (Life Technologies) and confirmed by immunoblotting using antibodies to N-HOIL1 (anti-N-terminal HOIL1; HPA024185; Sigma) and C-HOIL1 (anti-FLAG, clone M2, Sigma) cleavage products, respectively.

### Immunoblotting analysis of lymphocytes

The primary antibodies for immunoblotting and immunofluorescence confocal microscopy analyses were directed against: phospho-NF-κB p65 (Ser536; #3031), NF-κB p65 (#4764), IκBα (#9242), phospho-IκBα (ser32; 14D1), phospho-p65 (ser536; 93H1), phospho-ERK1/2 (Thr202/Tyr204; #9101) and β-actin (#3700 or #8457) all from Cell Signaling Technology; monoclonal antibodies to HOIL1 (clone E-2) and MALT1 (H-300) were from Santa Cruz Biotechnology, anti-N-terminal HOIL1 was from Sigma-Aldrich (HPA024185), and to the MALT1 N-terminal peptide was from Abcam (clone EP603Y). Anti-RIP1 antibody was from Cell Signaling Technologies (#4926). Anti-Otulin antibody was from Abcam (anti-FAM105B, ab151117). The secondary antibodies conjugated with infrared dye (IRDye 800 CW and 680 LT, 1:20,000) were from LI-COR Biosciences.

Immunoblotting analysis of PBMCs, T cells and immortalized B cells was as follows. Briefly, whole-cell lysates were prepared in modified RIPA lysis buffer (50 mM Tris-HCl, 150 mM NaCl, 2 mM EGTA and EDTA, and 1% TrionX-100, pH 7.5) with Halt protease and phosphatase inhibitors (Thermo Scientific). The lysed cells were incubated at 4 °C for 10 min followed by sonication. Proteins were separated by SDS–PAGE (10%), and then transferred onto a polyvinylidene difluoride membrane (Immobilon-FL, EMD Millipore) and incubated with primary antibodies for 18 h at 4 °C, and then secondary antibodies for 1 h at room temperature. Imaging was performed on a LI-COR Odyssey infrared imager (LI-COR Bioscience) and bands were quantified by densitometry using ImagJ freeware (NIH). Scanned images of immunoblots were cropped in the final figures for clarity and conciseness. Full images of all blots are shown in Supplementary Fig. 8.

### CBM complex and LUBAC cDNA cell transfections and analyses

HEK293FT cells (mycoplasma free) were grown in DMEM supplemented with 10% fetal calf serum, 2 mM L-glutamine, 100 U ml^−1^ penicillin and 100 μg ml^−1^ streptomycin (all from Amimed) and were transiently transfected using Roti-Fect (Carl Roth). The expression vectors for BCL10 and an active oncogenic mutant of CARD11 (Leu244Pro) have been described before^43^. The plasmids pDEST40-SHARPIN-V5-His, pDEST40-HOIP-V5-His and pDEST40-HOIL1-V5-His were from Invitrogen/Life Technologies. The expression vector for noncleavable HOIL1 pDEST40-HOIL1-Arg165Lys was made using QuikChange site-directed mutagenesis (Agilent Technologies). The vectors for wild type and catalytic inactive MALT1, pCMV-SPORT-Flag-His-S-MALT1 and pCMV-SPORT-Flag-His-S-MALT1 (Cys464Ala), respectively, and the N-terminally FLAG-tagged HOIL1 and its cleavage product analogues, N-HOIL1 (1-165) and C-HOIL1 (166-510), were made by standard procedures. pcDNA3.1-cIAP2-MALT1-Myc-His_6_ and pcDNA3.1-cIAP2-MALT1 (Cys464Ala)-Myc-His_6_ vectors were a kind gift of Dr Rudi Beyaert (University of Ghent, Belgium).

For cell lysis, cells were collected by centrifugation, washed in 1 × PBS and the cell pellets lysed in NP-40 lysis buffer (50 mM Tris-HCl, pH 7.5, 150 mM NaCl, 1.0% NP-40) supplemented with complete protease inhibitor cocktail and phosSTOP phosphatase inhibitor cocktail (Roche Life Science) and clarified by centrifugation. To preserve ubiquitin chains the cells were lysed by directly adding 1 × SDS reducing sample buffer to the washed cell pellets. After boiling, nuclear DNA was sheared by ultrasonication and proteins were separated on 4–12% Bis-Tris SDS–PAGE gradient gels (Life Technologies). Proteins were detected by immunoblotting using the following antibodies: anti-CYLD (E-10) and anti-BCL10 (H-197) from Santa Cruz Biotechnology; anti-MALT1 (MCA2801, clone 50) and anti-V5 (SV5-Pk1) from Serotec/AbD; anti-β-tubulin (AA2), anti-linear ubiquitin (MABS199), anti-K48-linked ubiquitin (Apu2) and anti-V5 (AB9732) from Merck-Millipore; anti-β-tubulin (tub2.1), anti-HOIL1 (HPA024185) and anti-Flag (M2) from Sigma-Aldrich; anti-RelB (C1E4), anti-CYLD (D1A10) and anti-CARD11 (1D12) from Cell Signaling Technology; anti-turboGFP (2H8) was from Origene; anti-HOIP (ab46322) from Abcam; and anti-linear ubiquitin (Lub9) from BioSensors. Horseradish peroxidase-coupled donkey anti-rabbit and sheep anti-mouse secondary antibodies were from GE Healthcare. Scanned images of immunoblots were cropped in the final figures for clarity and conciseness. Full images of all blots are shown in Supplementary Fig. 8.

### NF-κB promoter luciferase assays

HEK293FT cells were transfected with plasmid DNAs encoding for NF-κB promoter-driven firefly luciferase (κB_6_-Luc), a *Renilla* luciferase under the control of a constitutively active SV40 promoter, and combinations of the individual LUBAC components. At 36–48 h later, the cells were lysed in 100-μl passive lysis buffer (Promega) and the bioluminescence of the samples was measured using the Dual-Luciferase Reporter Assay System (Promega) and a Berthold TriStar luminometer. The relative luciferase activities were calculated after normalization of firefly luciferase activities to the activities of *Renilla* luciferase.

### Linear ubiquitin assays

For polyubiquitinated protein isolation, we used agarose-coupled TUBE2 (LifeSensors). Immortalized B cells (4 × 10^6^) and primary lymphocytes (1 × 10^7^) were seeded and rested at least 1 h, and treated with PMA/ionomycin with or without z-VRPR-fmk for various times and then lysed in modified RIPA buffer supplemented with Halt protease and phosphatase inhibitor cocktail (Thermo Scientific), and deubiquitinase inhibitors (5 mM *O*-phenanthroline and 50 μM PR619, LifeSensors). Cell lysates (550 μg) were mixed with 10 μl of pre-washed agarose-TUBE2 and incubated for 18 h at 4 °C. Agarose-TUBE2 was collected, washed and denatured in SDS–PAGE loading buffer. Samples were centrifuged and the supernatants were immunoblotted for total linear ubiquitin (LUB9 antibody, LifeSensors), NEMO (Santa Cruz Biotechnology; FL-419), RIP1 (Cell Signaling Technology; #4926) and K48-linked-polyubiquitin (Boston Biochem; #A-101). Scanned images of immunoblots were cropped in the final figures for clarity and conciseness. Full images of all blots are shown in Supplementary Fig. 8.

### Immunoprecipitation

For co-immunoprecipitation experiments, transfected cells were lysed in 50 mM Tris-HCl, pH 7.5, 150 mM NaCl and 0.5% NP-40 supplemented with complete protease inhibitor cocktail and phosSTOP phosphatase inhibitor cocktail (Roche Life Science). Cell lysates were incubated overnight with 1 μg precipitating antibody and 30 μl Protein G Sepharose (GE Healthcare). After three washing steps in the NP-40 lysis buffer, protein was eluted by boiling for 5 min in SDS sample buffer. Nonspecific binding controls showed that the tagged proteins did not bind beads alone or beads coupled with another antibody, for example, anti-FLAG antibody on beads controlled for V5-taged protein and vice versa.

### B-cell and antigen-presenting cell interactions

Experiments were performed as described in Freeman *et al.*^51^ The APCs were generated by transiently transfecting Cos7 cells using Lipofectamine 2000 (Invitrogen) with a plasmid encoding the surrogate Ag, which was a single-chain Fv containing the variable regions from the 187.1 rat anti-Igκ monoclonal antibody fused to the hinge and membrane-proximal domains of rat IgG1 and the transmembrane and cytoplasmic domains of H-2K (ref. 61). After 24 h, transfected Cos7 APCs were detached using enzyme free dissociation buffer (0.5 mM EDTA, 100 mM, NaCl, 1 mM glucose, pH 7.4) and 1.5 × 10^4^ cells were plated on 18-mm glass coverslips that had been coated with 5 μg ml^−1^ fibronectin. The APCs were cultured for 12 h in DMEM plus 10% FBS supplemented with 1% L-glutamine, 1% Na pyruvate and 0.05% penicillin/streptomycin to allow cells to spread and flatten.

Murine splenic B cells were isolated from C57BL/6J mice (9-week 4-day male, and 19-week 4-day male) (animal protocols were approved by the University of British Columbia Animal Care Committee) using a B-cell isolation kit (Stemcell Technologies) to deplete non-B cells. B cells (2 × 10^6^) were resuspended in 0.4 ml modified HEPES-buffered saline (25 mM sodium HEPES, pH 7.2, 125 mM NaCl, 5 mM KCl, 1 mM CaCl_2_, 1 mM Na_2_HPO_4_, 0.5 mM MgSO_4_, 1 mg ml^−1^ glucose, 2 mM glutamine, 1 mM sodium pyruvate, 50 μM 2-mercaptoethanol) supplemented with 2% FBS and then incubated with 75 μM z-VRPR-fmk or an equivalent volume of H_2_O for 30 min at 37 °C. The B cells (1 × 10^6^ per coverslip) were then added to the Cos7 APCs and allowed to attach for 10–30 min at 37 °C before fixing the cells with 4% paraformaldehyde.

Before staining, the cells were permeabilized with 0.1% Triton X-100 and then blocked with 2% BSA, 10% goat serum and 25 μg ml^−1^ of the 2.4G2 monoclonal antibody, which blocks Fc receptors on the B cells. The cells were then stained with AlexaFluor 568-conjugated goat anti-rat IgG (H+L chain-reactive; Life Technologies, catalogue number A11077) to visualize the anti-Igκ surrogate Ag, and with a mouse IgG1 anti-linear polyubiquitin (LUB9) monoclonal antibody (LifeSensors, catalogue # AB130, the same antibody used to probe the TUBEs assays)), the same antibody was used to probe the TUBE assay blots, followed by staining with an AlexaFluor 647-conjugated goat anti-mouse IgG1 (γ1 H chain-specific; Life Technologies, catalogue number A21240) secondary antibody and AlexaFluor 488 Phalloidin (Life Technologies catalogue # A12379) to visualize F-actin. Naive primary B cells, which express IgM and IgD, but not IgG, were not stained to any significant extent by the goat anti-mouse IgG1 secondary antibody in the absence of primary antibody. Coverslips were mounted using ProLong Diamond Antifade Mountant (Life Technologies, catalogue #P36961).

Cells were imaged using spinning disk confocal microscope system (3i—Intelligent Imaging Innovations) with a Zeiss Axiovert 200M microscope with a × 100 numerical aperture 1.45 oil Pan-Fluor objective and QuantEM 512SC Photometrics camera. All images were acquired using identical settings and analysed using FIJI and MATLAB software. Optical *z*-slices were imaged through the cell at 0.2-μm intervals. To produce the side view in Fig. 8b. FIJI software was used to reslice the *z*-stacks at 0.2-μm intervals. A centre slice was picked for images. MATLAB software was used to analyse images for fluorescence intensity. Fluorescence signals for linear ubiquitin and antigen (single-chain anti-Igκ) were measured at the contact site between the B cell and the Cos7 APCs. For cells where the contact site between B cell and the APC was not in one focal plane could not be analysed and were excluded.

To compute fluorescence intensities, each image was first filtered using Laplacian of Gaussian (LoG) filters at a range of scales (scale=square of Gaussian s.d.). Specifically, the Gaussian s.d.'s used were every value in the range from 0.08 to 0.48 μm at intervals of 0.04 μm. To threshold the image for noise, the median absolute deviation of pixel values was computed and used to estimate (multiplying by 1.4826) the s.d. For each filtered image, the threshold was set at six noise s.d.'s above the mean pixel intensity. Finally, a mask generated by retaining all pixels present at any scale after thresholding LoG filtered images was applied to the original image and all pixel values within the mask were summed to yield the total fluorescence intensity. This value was normalized to the total surrogate antigen fluorescence intensity for the same cell.

### N-terminal 10-plex TMT TAILS and shotgun proteomics

The B-cell N-terminome and proteome from the patient (*n*=3), brother (*n*=1) and mother (*n*=1), with and without PMA/ionomycin stimulation for 2 and 4 h, were analysed by TAILS and shotgun proteomics (*N*=2)^39^,^40^. B-cell lysate protein was prepared by snap-freezing cells in hypotonic lysis buffer (10 mM HEPES, pH 7.5 supplemented with protease inhibitor cocktail, Roche). After removal of cell debris by centrifugation, 200 μg per condition was precipitated by chloroform–methanol and dissolved in 100 μl labelling buffer (2 M guanidine hydrochloride and 200 mM HEPES, pH 8.0). Proteins were reduced with TCEP (10 mM, 30 min) and cysteine residues alkylated with iodoacetamide (25 mM, 30 min in the dark). For TMT labelling of proteins, 10-plex TMT labels (0.8 mg each, Pierce) were dissolved in a volume of dimethylsulphoxide equal to the total reaction mixture at that point and added 1:1 to the proteome samples for 1 h in the dark. Unreacted TMT label was quenched with ethanolamine (50 mM, 1 h) and all 10 TMT-labelled samples were combined. The samples were cleaned up by precipitation with ice-cold acetone/methanol (9:1) for 2 h and precipitates resuspended in 50 mM HEPES buffer, pH 8.0 and then digested with trypsin (Trypsin Gold, Promega) at 37 °C for 16 h.

After trypsinization, 1% of the sample was desalted on a C_18_ StageTip and analysed for shotgun proteomics by liquid chromatography (LC) MS/MS. The rest of the sample was enriched for N-terminal peptides as follows. Tryptic peptides displayed unblocked N-terminal α-amines enabling their removal by coupling to a hyperbranched polyglycerol polyaldehyde-derivatized polymer (HPG-ALD; Flintbox Innovation Network: http://flintbox.com/public/project/1948/) by reductive amination (10 mg polymer and 20 mM sodium cyanoborohydride, pH 6.5) at 37 °C for 16 h. After quenching unreacted aldehydes with ethanolamine (100 mM, 20 mM fresh sodium cyanoborohydride) for 1 h, the TMT, acetyl- and pyroglutamate-blocked N-terminal peptides were separated from the polymer by ultrafiltration using a 10-kDa molecular weight cut-off (MWCO) spin filter (Amicon). The filtrate containing the N terminome (TAILS sample) was desalted on a C_18_ StageTip and analysed by LC-MS/MS and LC-MS/MS/MS.

### Mass spectrometry

Dried TAILS and preTAILS shotgun samples were resuspended in 5% acetonitrile and 0.1% TFA. An Easy-nLC 1000 (Thermo Fisher Scientific, San Jose, CA) was employed to perform reverse-phase nano-high-performance liquid chromatography peptide separation and introduction into an Orbitrap Fusion Tribrid mass spectrometer (Thermo Fisher Scientific) using 0.1% formic acid in water as mobile phase A and 0.1% formic acid in acetonitrile as mobile phase B. Approximately 1 μg of sample in 2 μl of solution was loaded onto a 2 cm Acclaim PepMap100 trap column to perform online desalting followed by transfer to an EASY-Spray PepMap 50 cm × 75 μm C_18_ Column (Thermo Fisher Scientific, Bellefonte, PA) for analytical separation. Peptides were eluted using a gradient of 5–25% mobile phase B over 180 min followed by 25–40% B over an additional 30 min at a constant flow rate of 300 nl min^−1^. The column was regenerated at 95% B for 10 min.

All samples were analysed in duplicate as technical replicates. Mass spectrometry data were collected using top speed mode and a maximum cycle time of 3 s. An Orbitrap full MS scan was acquired from 350 to 1,600 *m*/*z* using a resolution of 120,000 full width at half maximum at 200 *m*/*z* and an ion target of 2 × 10^5^. The most abundant monoisotopically resolved precursors within a charge range of +2 to +9 were selected using a 2 AMU quadrupole isolation width for MS/MS product by higher-energy collisional dissociation (HCD) at 40% normalized collision energy followed by detection in the Orbitrap at a resolution of 60,000 with an ion target of 10^5^. A fixed first mass of 110 *m*/*z* was used for MS/MS scans.

SPS^62^ mode acquisition was executed using an Orbitrap full MS scan from 350 to 1,600 *m*/*z* with a resolution of 120,000 full width at half maximum in top speed mode with a 3 s maximum cycle time as follows. The most abundant monoisotopically resolved precursors with charges from +2 to +9 were selected by quadrupole isolation at 2 AMU for MS/MS collision-induced dissociation (CID) fragmented in the linear ion trap at 30% normalized collision energy. Up to 10 of the most intense products from the MS/MS spectra were selected, coisolated and fragmented together in the HCD cell at 55% normalized collision energy with subsequent detection in the Orbitrap at 60,000 resolution with a scan range of 100–500 *m*/*z* and an ion target of 10^5^. A variation of this method employing charge-dependent MS/MS fragmentation using either CID or electron-transfer dissociation (ETD) in the ion trap was also performed.

### Proteomics data analysis

All data analysis was conducted using Thermo Scientific Proteome Discoverer 2.0 and the integrated Byonic v1.4 search node. Ion trap spectra were searched with mass tolerances of 10 p.p.m. for the precursor ion and 15 p.p.m. (Orbitrap) or 0.6 Da (ion trap) for fragment ions. Quantification was performed using the MS/MS spectra for the Orbitrap MS/MS HCD method and the MS/MS/MS spectra for the SPS method. Technical replicates were searched together. After initial assessment of labelling efficiency, carbamidomethylation (+57.021 Da) of cysteine and TMT isobaric labelling (+229.162 Da) of lysine were set as static modifications, whereas TMT labelling of the protein N termini, acetylation of protein N termini (+42.011), formation of pyroglutamate from glutamine on peptide N termini (−17.027), deamidation of asparagine and glutamine (+0.984 Da) and methionine oxidation (+15.996 Da) were considered dynamic modifications. Because of the TMT labelling of lysine residues, which trypsin does not recognize, data were searched using semi-specific ArgC search parameters with up to two missed cleavages. Spectra were searched against the complete Swiss-Prot human database (release 2013_08) at a 1% protein level FDR. Resulting peptide lists were filtered for peptides identified with a probability >0.99.

Working peptide lists for baseline ratio determination in the TAILS experiments were filtered for peptides containing N-terminal TMT, acetyl or pyroglutamate. Reporter ion ratios were calculated as heterozygous control (*MALT1*^*+/mut*^ brother or mother)/average homozygous patient (*MALT1*^*mut/mut*^; *n*=3) per experiment and per condition, yielding eight separate distribution lists. Significant outlier cutoff values were determined after log(2) transformation by boxplot-and-whiskers analysis using the BoxPlotR tool^63^. Venn diagrams were created using the BioVenn web application^64^.

### Substrate winnowing

Candidate MALT1 substrates were selected by a new substrate winnowing strategy (Supplementary Fig. 3) and by the following criteria. A substrate was identified only if the neo-N-terminal peptide was internal, that is, not starting at a recognized protein start or processing site, and was N-terminally blocked by a TMT label. Moreover, the neo-N-terminal peptides must be significantly more abundant in samples from the heterozygous controls compared with the MALT1-deficient patient and with the peptide-spectrum match (PSM) ratio higher than the 75th percentile+1.5 × interquartile range; the neo-N-terminal peptide must have increased abundance in PMA/ionomycin-stimulated samples compared with the vehicle control for the same subject; where the protein natural N terminus was also identified the neo-N-terminal peptide to natural N-terminal peptide ratio was high to ensure that cleavage was not merely increased concomitantly with increased total protein after stimulation of between cells; the neo-N-termini were reproducibly identified in two or more TAILS samples and by ≥3 spectra; finally, the cleavage site needed to conform to the strict MALT1-cleavage site specificity of an arginine at P1 and was cleaved in biochemical *in vitro* cleavage assays.

## Additional information

**How to cite this article:** Klein, T. *et al.* The paracaspase MALT1 cleaves HOIL1 reducing linear ubiquitination by LUBAC to dampen lymphocyte NF-κB signalling. *Nat. Commun.* 6:8777 doi: 10.1038/ncomms9777 (2015).

## Supplementary Material

Supplementary Information Supplementary Figures 1-8
