# Supplementary material for: The paracaspase MALT1 cleaves HOIL1 reducing linear ubiquitination by LUBAC to dampen lymphocyte NF-κB signalling
Source: Nat Commun. 2015 Nov 3;6:8777. doi: 10.1038/ncomms9777 (PMC4659944; doi:10.1038/ncomms9777)
Supplement: Supplementary Information — Supplementary Figures 1-8 [file ncomms9777-s1.pdf]

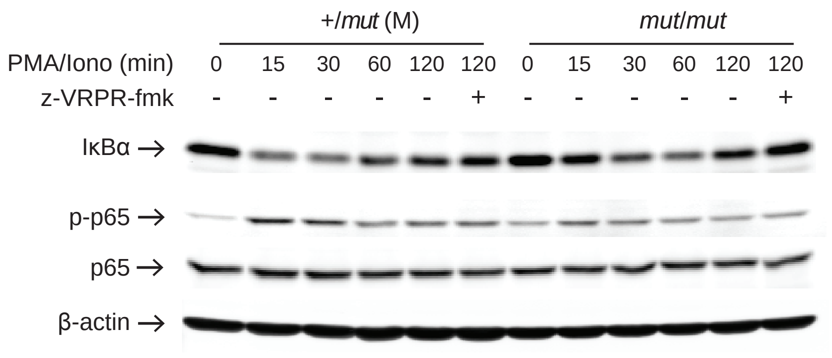

Supplementary Figure 1. Immunoblots from a single representative experiment (*N* = 12) showed a decreased NF-κB response, as quantified by degradation of IκBα and phosphorylation of p65 to p-p65 in immortalized B cells from the patient (*mut*/*mut*) compared to the heterozygous mother (+/*mut* (M)).

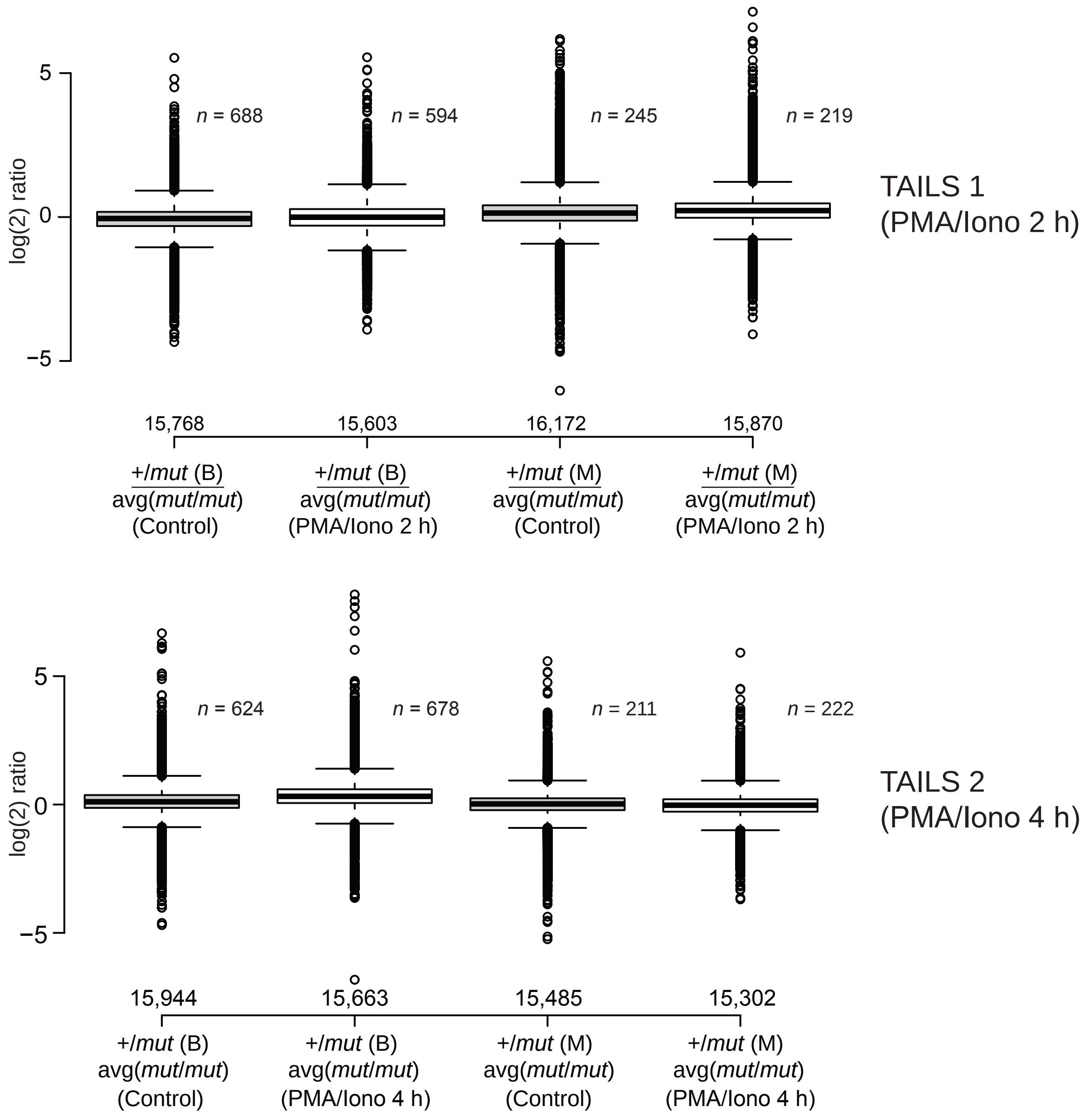

Supplementary Figure 2 Boxplot-and-whiskers analysis of all quantifyable peptide-spectrum-matches was used to determine statistical cut-off values for log (2) ratios of heterozygote control vs. patient samples.

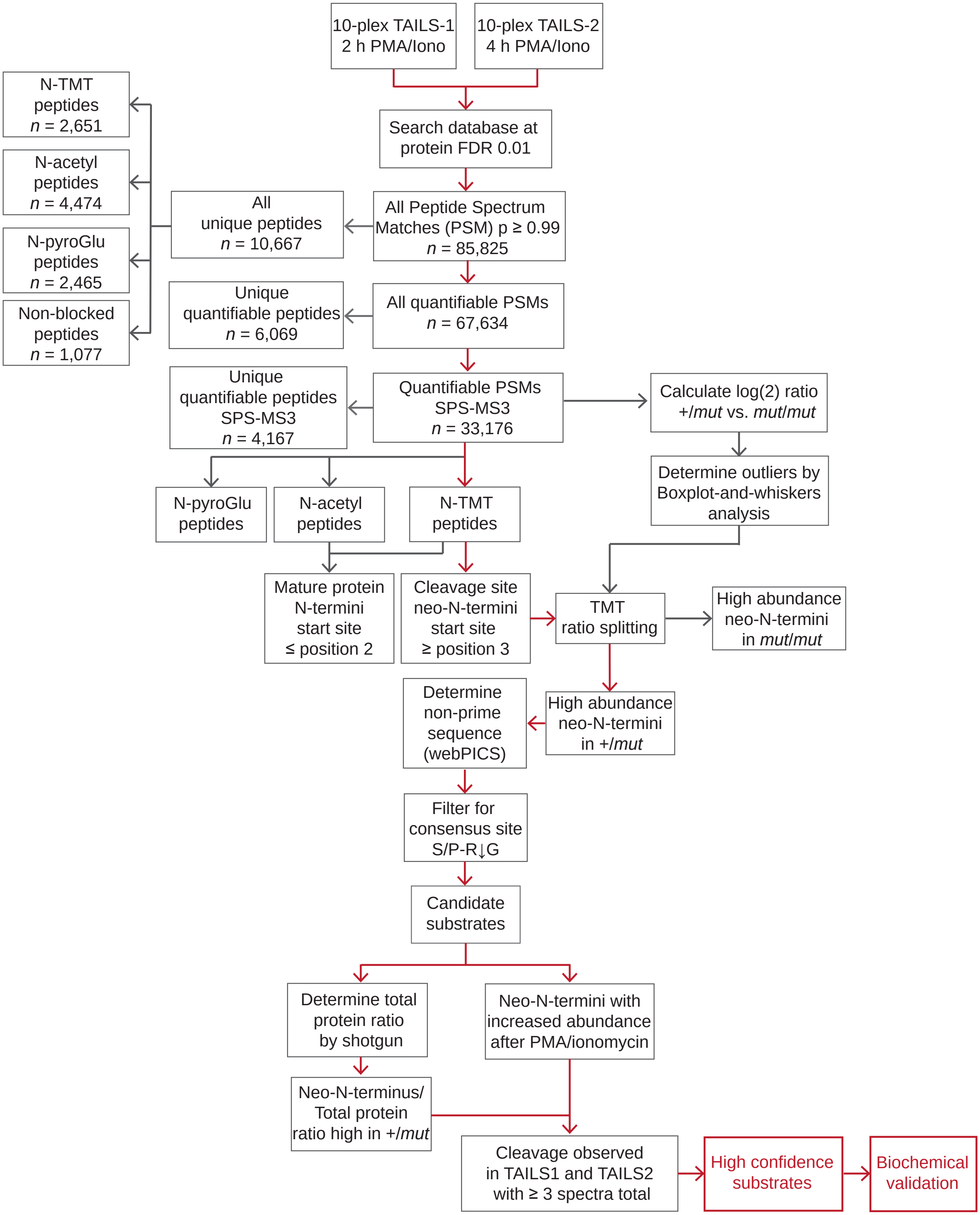

Supplementary Figure 3 Data winnowing strategy for identification of novel high confidence MALT1 substrates. Data from TAILS analyses (*N* = 2) of homozygous (*mut*/*mut*, *n* = 3) and heterozygous (+/*mut*, *n* = 2) immortalized B cells under control and PMA/ionomycin stimulated conditions was filtered to discover MALT1 substrates with high confidence.

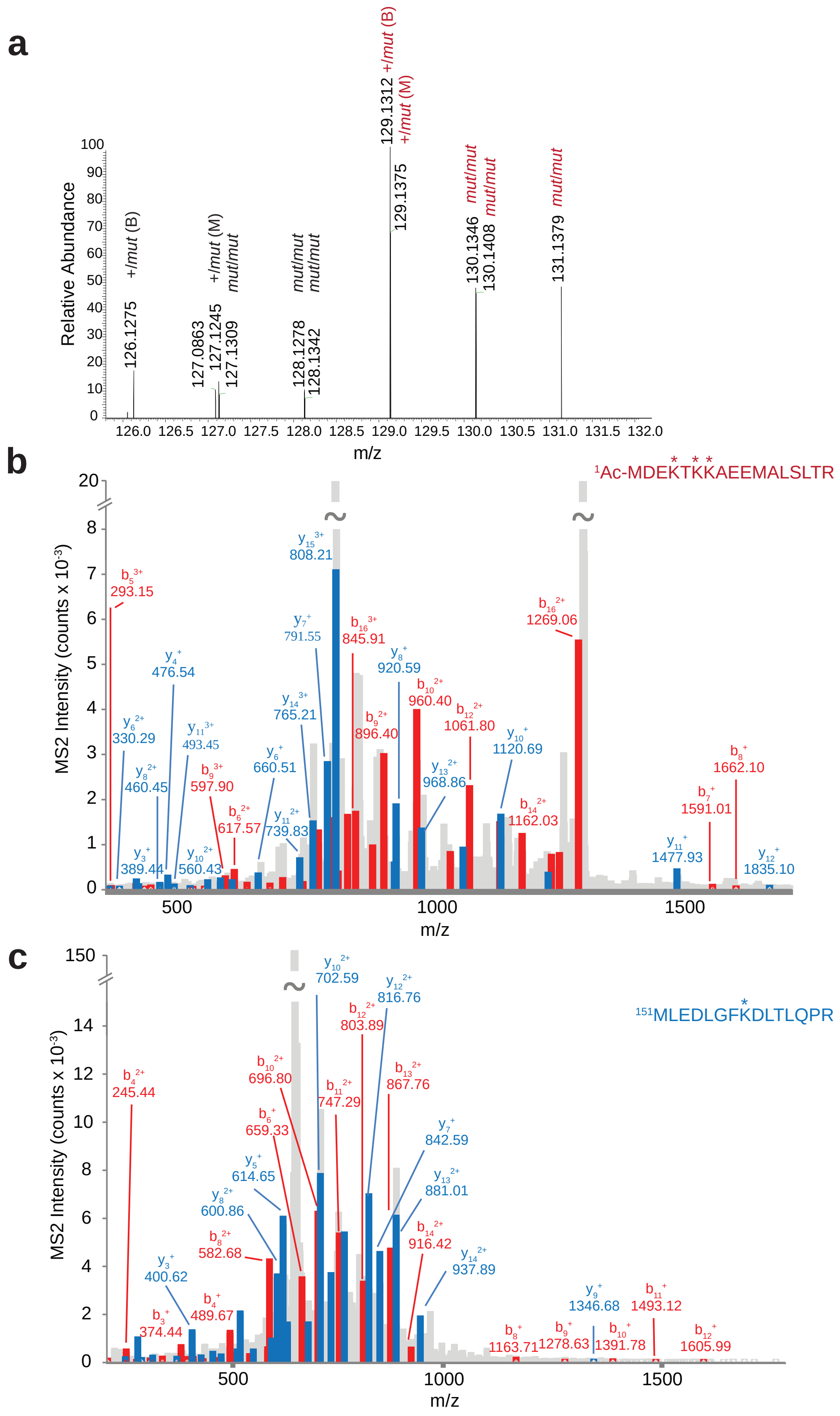

Supplementary Figure 4. Mass spectrometry data supporting the discovery of HOIL1 as a substrate of MALT1. (**a**) 10-plex TMT reporter ion spectrum for the neo-N-terminal HOIL1 peptide identified by TAILS. (**b**) Byonic spectral match for the natural N-terminal peptide of HOIL1 identified by TAILS. (**c**) Byonic spectral match for the internal tryptic nonprime side cleaved HOIL1 peptide identified by shotgun proteomics. *, TMT-labeled amino acid residue. Ac, acetylated.

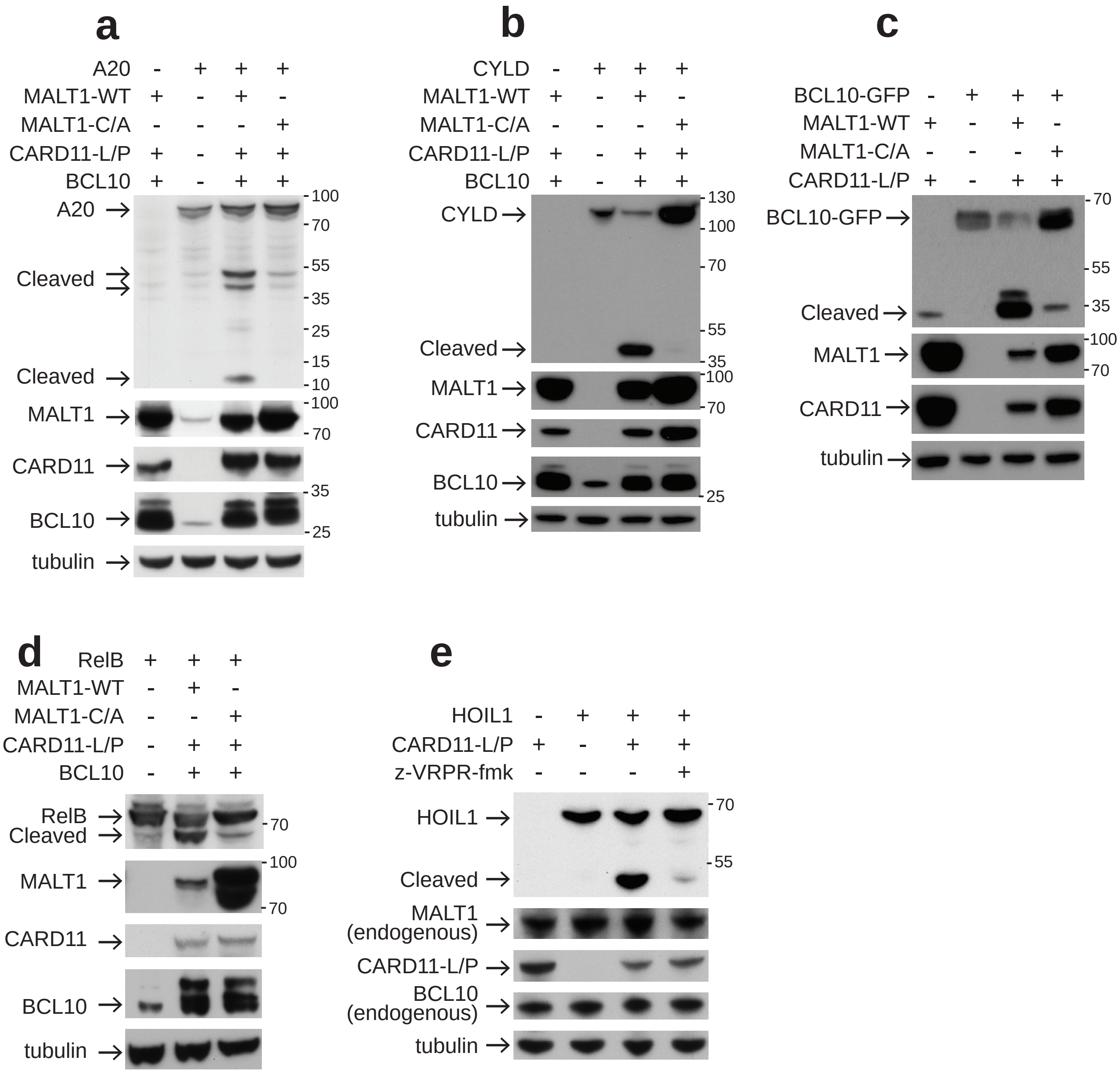

Supplementary Figure 5 Validation of the cotransfection system in HEK293FT cells for intracellular cleavage of MALT1 substrates. To validate HOIL1 cleavage by MALT1 in the cellular context we established a transfection system in which an active CBM complex was assembled in HEK293FT cells by coexpressing MALT1 with BCL10 and with an active oncogenic mutant of CARD11 (Leu244Pro).
The system was validated by demonstrating intracellular cleavage of cotransfected known MALT1 substrates A20 (*N* = 4) (**a**), CYLD (*N* = 3) (**b**), BCL10 (*N* = 8) (**c**) and RelB (*N* = 4) (**d**). BCL10 was expressed as a C-terminal green fluorescent protein tagged construct in order to visualize the scissile bond five residues from the C-terminus. Cleavage of substrates was absent or largely abrogated when a catalytically inactive mutant of MALT1 (Cys464Ala) was transfected in place of wild-type MALT1. (**e**) Endogenous levels of MALT1 and BCL10 in HEK293FT cells were sufficient to also form an active CBM complex with transfected CARD11-L/P, leading to MALT1 paracaspase-dependent cleavage of cotransfected HOIL1 that could be inhibited with z-VRPR-fmk (*N* = 2). Molecular weight markers positions as indicated.

Supplementary Figure 6

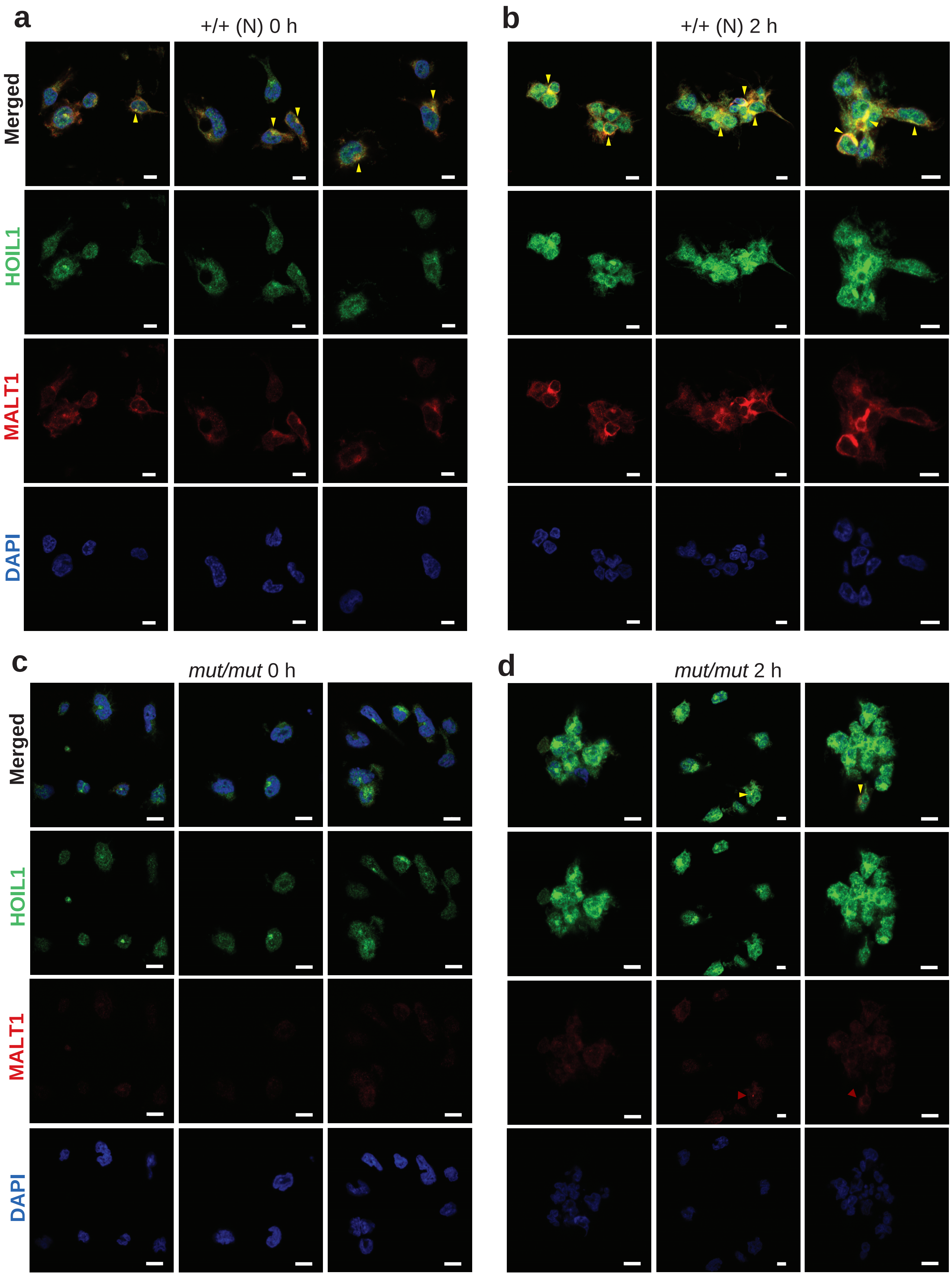

Supplementary Figure 6 Visualisation of HOIL1 and MALT1 interaction in B cells. Confocal microscopy of HOIL1 and MALT1 showed co-localization (yellow arrows) in (**a**) non-prestimulated (0 h) and (**b**) anti-IgG-prestimulated (2 h) immortalized B cells from a normal donor (+/+ (N)). (**c**,**d**) In patient (*mut/mut*) B cells the unstable MALT1 (Trp580Ser) levels were low and showed little colocalization with HOIL1. Monoclonal antibodies to HOIL1 (green) and MALT1 (red) were counterstained with DAPI nuclear stain (blue); colocalized proteins are seen as yellow upon merging. Click-to-zoom high resolution images. Scale bar, 10 μm.

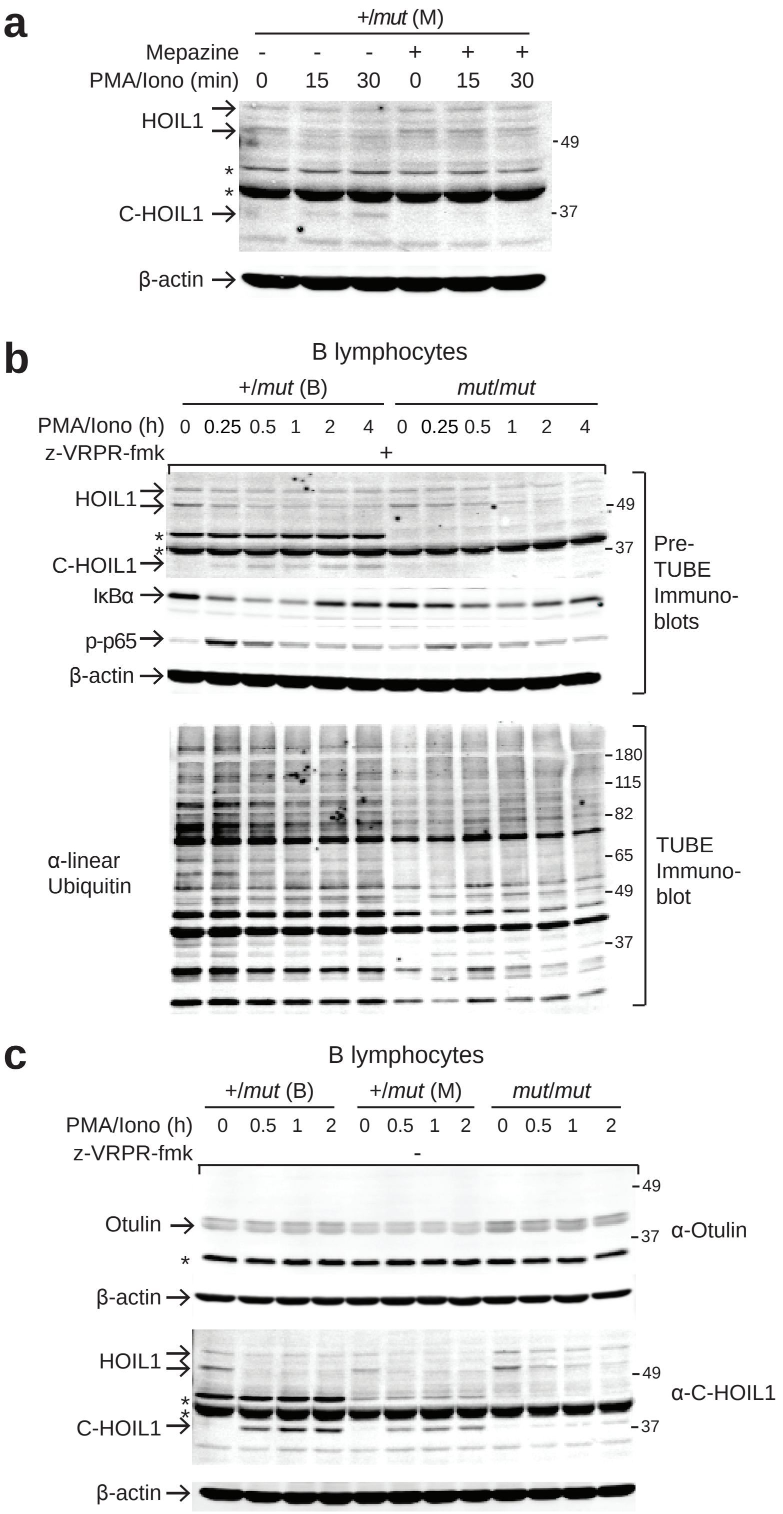

Supplementary Figure 7 (**a**) Immunoblot showing pretreatment of mother (+/*mut* (M)) immortalized B cells with MALT1 inhibitor Mepazine blocked cleavage of HOIL1 *N* = 3. *, non-specific band. β-actin, loading control. (**b**) Immunoblots showing that preincubation of immortalized B cells from the brother (+/*mut* B) with MALT1 inhibitor z-VRPR-fmk inhibited HOIL1 cleavage and abrogated the rapid decrease in total linear ubiquitinated conjugates as shown after TUBE-pull down. NF-κB was activated normally as shown by immunoblotting for p-p65 and IκBα. Patient cells (*mut*/*mut*) were unaffected compared to the untreated cells shown in **Fig. 6*a*** (*N* = 2). β-actin, loading control. (**c**) Immunoblots showing protein abundance of the specific linear ubiquitin-targeting deubiquitinase otulin remain unchanged upon PMA/ionomycin stimulation in EBV-B cells from heterozygous brother (+/*mut* B), mother (+/*mut* M) and the patient (*mut*/*mut*). β-actin, loading control. *, Consistently observed non-specific bands (*N* = 2).

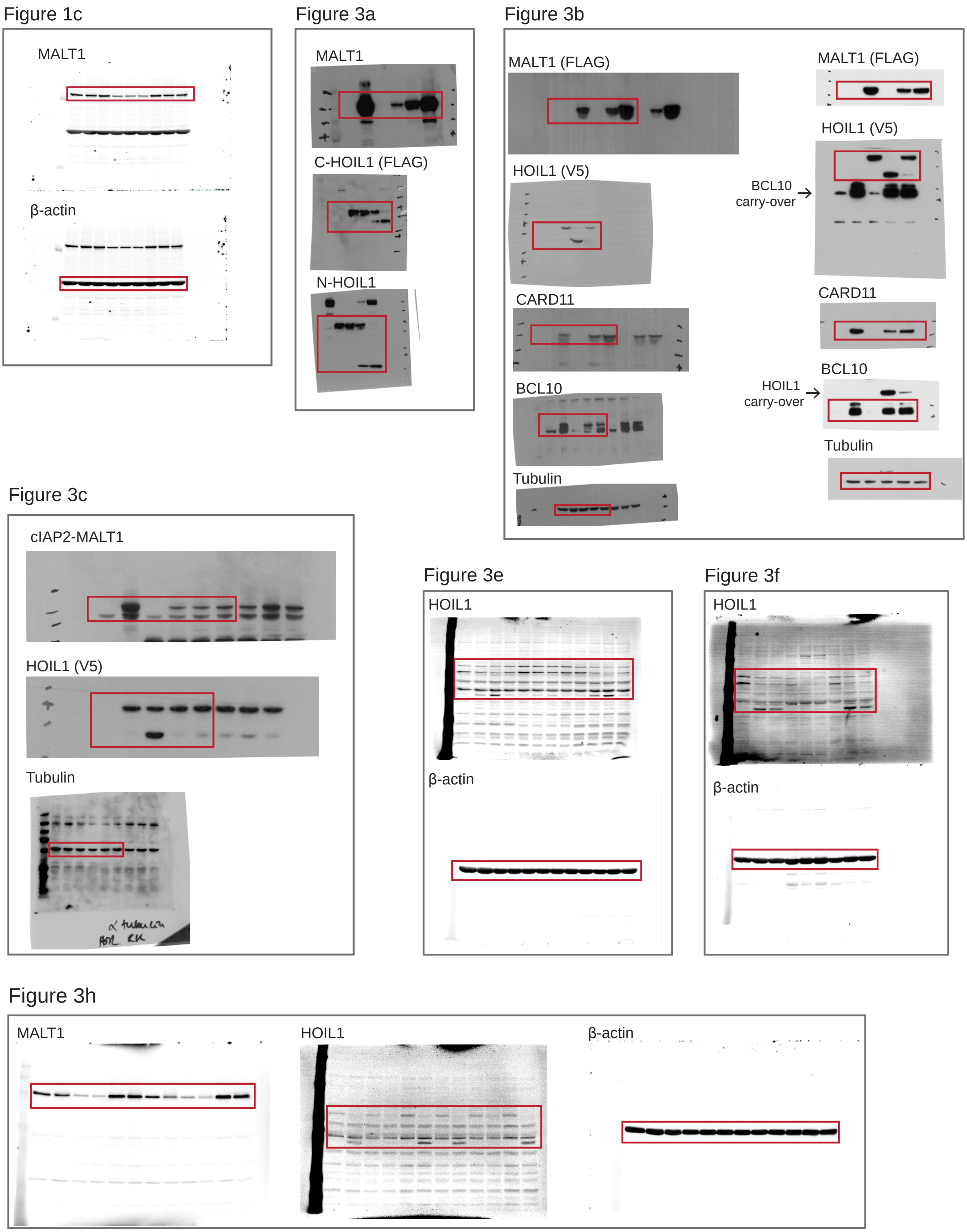

Supplementary Figure 8*a*: Full images of immunoblots shown in Figures 1 and 3. Red boxes show approximate image used for presentation.

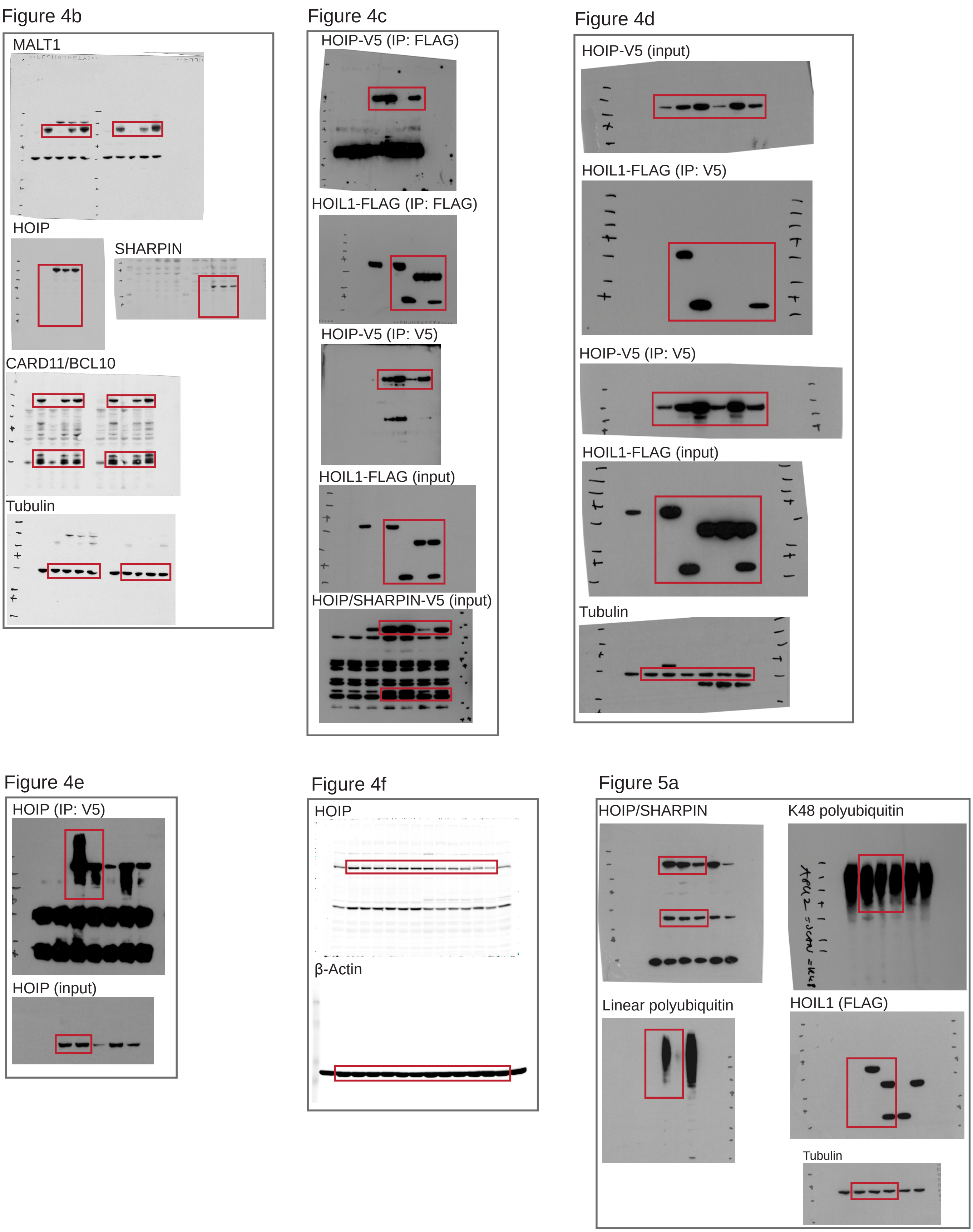

Supplementary Figure 8*b*. Full images of immunoblots shown in Figures 4*b-f* and 5*a*. Red boxes show approximate image used for presentation.

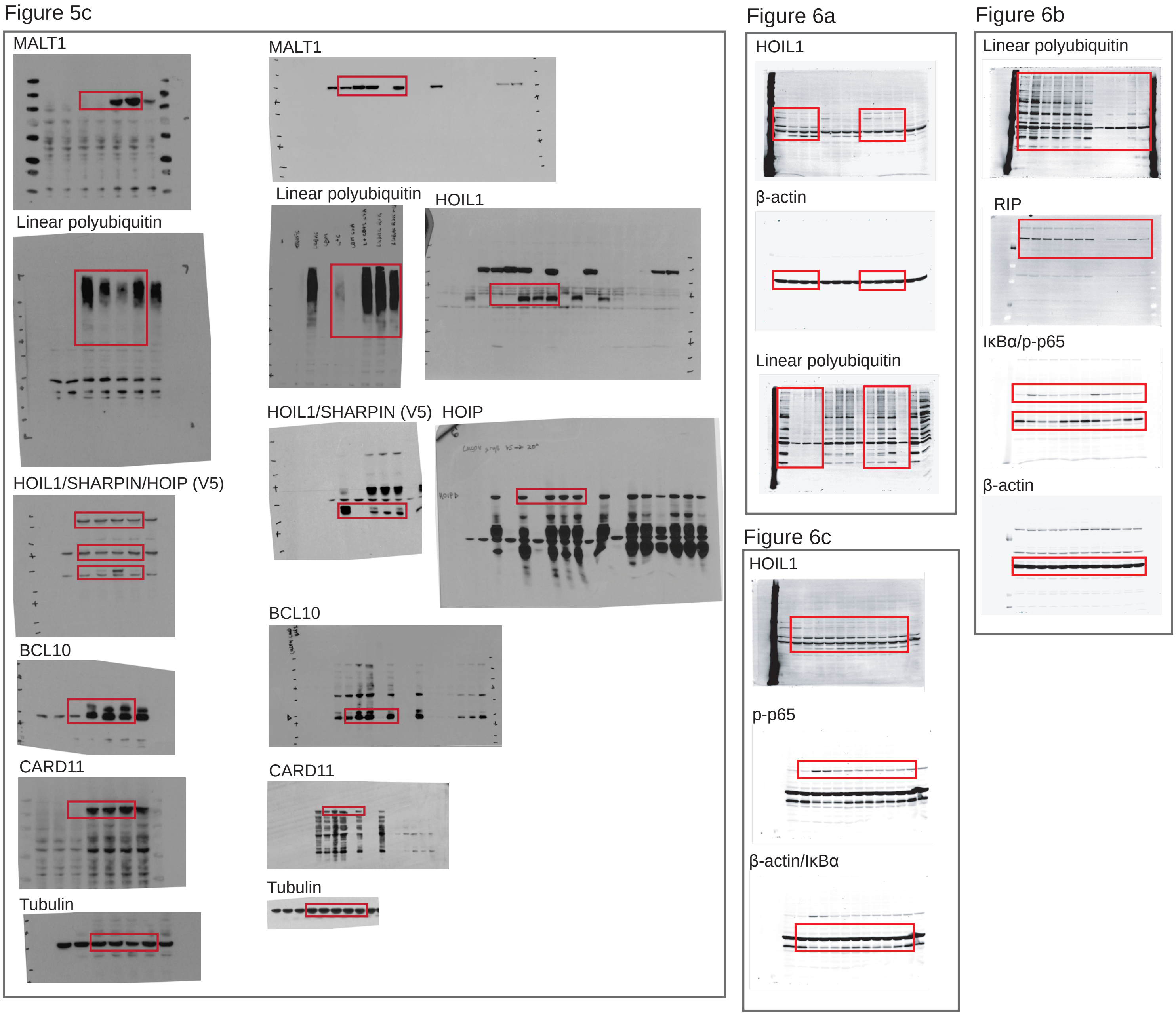

Supplementary Figure 8*c*. Full images of immunoblots shown in Figures 5*c* and 6*a*-*c*. Red boxes show approximate image used for presentation.

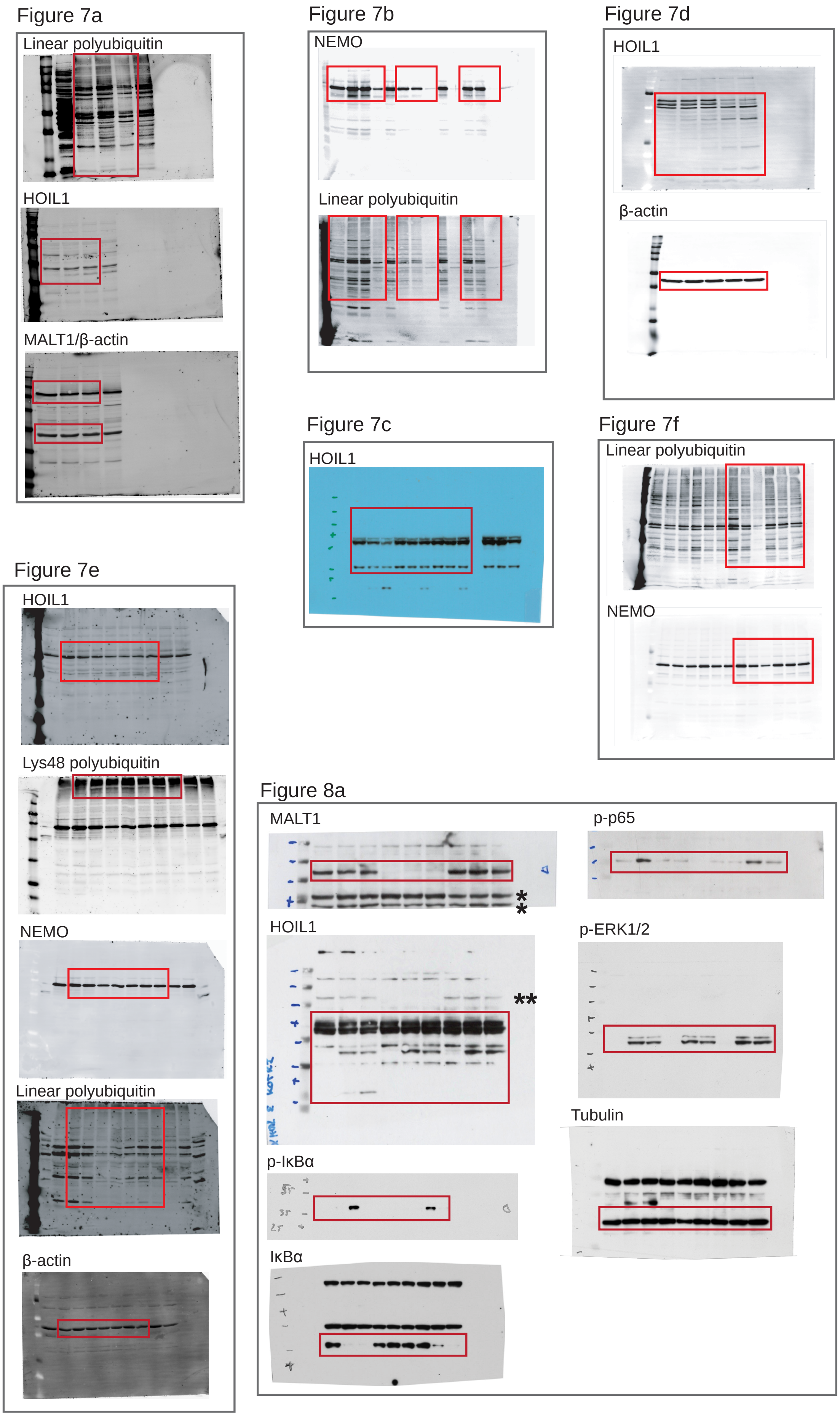

Supplementary Figure 8*d*. Full images of immunoblots shown in Figures 7*a-e* and 8*a*. Red boxes show approximate image used for presentation. In Figure 8a blots, *: HOIL1 carry over; ** MALT1 carry over.

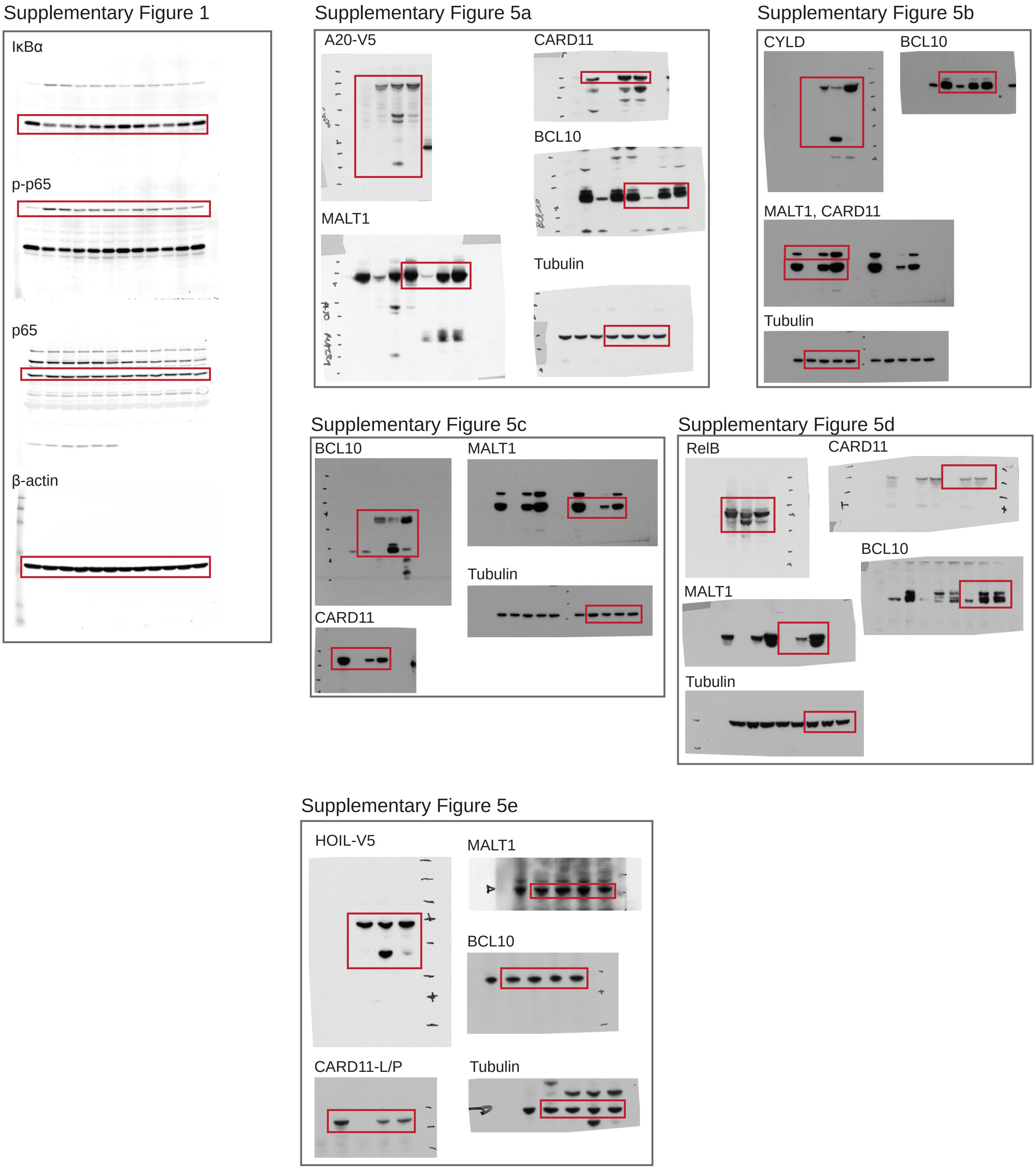

Supplementary Figure 8e. Full images of immunoblots shown in Supplementary Figures 1 and 5. Red boxes show approximate image used for presentation.

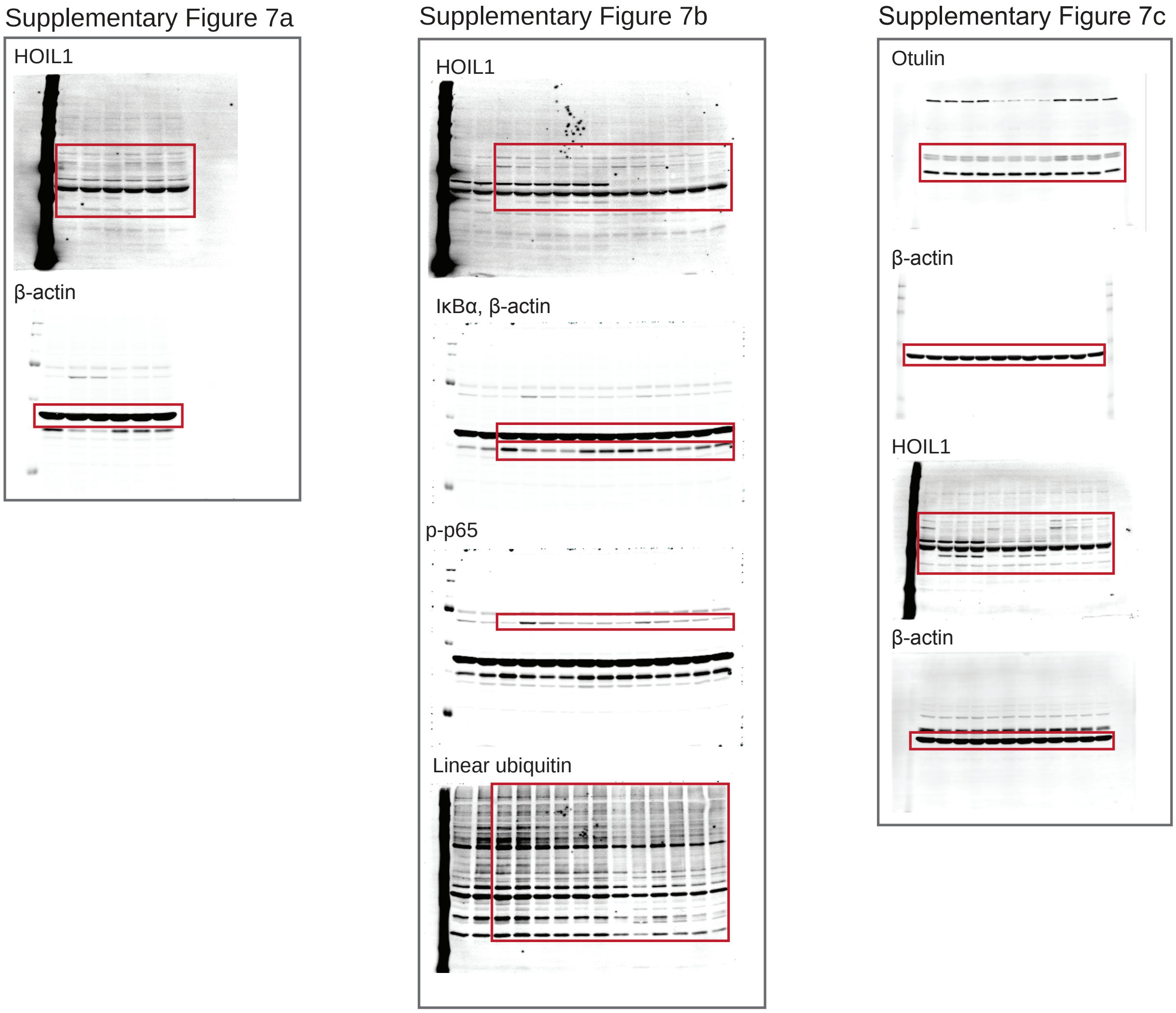

Supplementary Figure 8*f*. Full images of immunoblots shown in Supplementary Figure 7. Red boxes show approximate image used for presentation.
